# High Versus Low Ligation of the Inferior Mesenteric Artery in Colorectal Cancer Surgery: A Systematic Review and Meta-Analysis

**DOI:** 10.3390/medicina58091143

**Published:** 2022-08-23

**Authors:** Kwangmin Kim, Sanghyun An, Myung Ha Kim, Jae Hung Jung, Youngwan Kim

**Affiliations:** 1 Department of Surgery, Yonsei University Wonju College of Medicine, Wonju 26426, Korea; 2 Trauma Center, Wonju Severance Christian Hospital, Wonju 26426, Korea; 3 Division of Colorectal Surgery, Yonsei University Wonju College of Medicine, Wonju 26426, Korea; 4 Yonsei Wonju Medical Library, Yonsei University Wonju College of Medicine, Wonju 26426, Korea; 5 Department of Urology, Yonsei University Wonju College of Medicine, Wonju 26426, Korea; 6 Center of Evidence Based Medicine, Institute of Convergence Science, Yonsei University, Seoul 03722, Korea

**Keywords:** colorectal cancer, inferior mesenteric artery, high ligation, low ligation

## Abstract

*Background and Objectives*: This study aimed to compare the effects of high ligation (HL) versus low ligation (LL) in colorectal cancer surgery. *Materials and Methods*: We performed a comprehensive search using multiple databases (trial registries and ClinicalTrials.gov), other sources of grey literature, and conference proceedings, with no restrictions on the language or publication status, up until 10 March 2021. We included all parallel-group randomized controlled trials (RCTs) and considered cluster RCTs for inclusion. The risk of bias domains were “low risk,” “high risk,” or “unclear risk.” We performed statistical analyses using a random-effects model and interpreted the results according to the *Cochrane Handbook for Systematic Reviews of Interventions*. We used the GRADE guidelines to rate the certainty of evidence (CoE) of the randomized controlled trials. *Results*: We found 12 studies (24 articles) from our search. We were very uncertain about the effects of HL on overall mortality, disease recurrence, cancer-specific mortality, postoperative mortality, and anastomotic leakage (very low CoE). There may be little to no difference between HL and LL in postoperative complications (low CoE). For short-term follow-up (within 6 months), HL may reduce defecatory function (constipation; low CoE). While HL and LL may have similar effects on sexual function in men, HL may reduce female sexual function compared with LL (low CoE). For long-term follow-up (beyond 6 months), HL may reduce defecatory function (constipation; low CoE). There were discrepancies in the effects regarding urinary dysfunction according to which questionnaire was used in the studies. HL may reduce male and female sexual function (low CoE). *Conclusions*: We are very uncertain about the effects of HL on survival outcomes, and there is no difference in the incidence of postoperative complications between HL and LL. More rigorous RCTs are necessary to evaluate the effect of HL and LL on functional outcomes.

## 1. Introduction

Colorectal cancer accounts for approximately 10.2% of all cancers and is the third-most-common cancer in terms of incidence and the second-most-common cause of cancer-related mortality [1]. Left-sided colorectal cancer accounts for up to two-thirds of all colorectal cancers [2]. Most patients can be treated with radical surgery with or without perioperative chemotherapy and radiotherapy. Surgery has been recommended as the gold-standard treatment for colorectal cancer.

The removal of the tumor and wide resection of the colonic mesentery with vascular ligation have been the standard techniques for the surgical treatment of left-sided colon cancer and rectal cancer [3]. However, currently, there is no worldwide consensus on the optimal level of arterial ligation in terms of oncological outcomes, postoperative morbidity, and functional outcomes. Thus, most surgeons determine the level of ligation based on experience.

In 1908, Moynihan and Miles first introduced two different techniques for the ligation of the inferior mesenteric artery (IMA): high ligation (HL) and low ligation (LL) [4]. HL refers to the ligation of the IMA immediately after it branches off the anterior surface of the abdominal aorta, whereas LL refers to ligation at the level of the superior rectal artery, preserving the left colic artery (LCA).

When determining the level of IMA ligation, oncologic outcomes, functional outcomes, and technical safety should be considered. In general, HL of the IMA is technically difficult, may enable more radical lymphadenectomy [5,6], and may lead to adverse functional outcomes because autonomic nerves around the origin of the IMA may be damaged. In contrast, LL of the IMA may provide an abundant blood supply to the proximal end of the anastomotic site, reduce the risk of autonomic nerve injury, and result in less radical lymph node dissection [7,8].

Therefore, we hypothesized that HL of the IMA may be beneficial for patients’ survival compared with LL, because more radical lymphadenectomy may be possible. In addition, LL of the IMA may be better than HL in terms of functional outcomes and postoperative complications due to the preservation of autonomic nerve functions, abundant blood supply, and less radical lymph node dissection.

In this study, we tried to analyze important patient outcomes, such as survival outcomes, postoperative complications, and anastomotic leakage. In addition, in particular, functional outcomes, such as defecatory dysfunction, urinary dysfunction, and sexual dysfunction, were assessed in our review to determine which level of ligation is superior in terms of functional impairment.

## 2. Materials and Methods

### 2.1. Literature Search

This systematic review and meta-analysis was performed according to the protocol published by PROSPERO (registration number: CRD42021241241). This systematic review follows PRISMA guidelines (Tables S1 and S2).

We performed a comprehensive search of several databases, including MEDLINE, EMBASE, Cochrane Library, Scopus, Web of Science, Latin American and Caribbean Health Sciences Literature, and other resources, such as ClinicalTrials.gov (www.clinicaltrials.gov/: accessed on 10 March 2021), the World Health Organization International Clinical Trials Registry Platform search portal (apps.who.int/trialsearch/: accessed on 10 March 2021), and OpenGrey (www.opengrey.eu/: accessed on 10 March 2021). Table S3 presents the search strategy for each database. We also searched the reference lists of the selected studies for Supplemental Studies and contacted their authors for reports of unpublished or published studies, including new or additional studies, or works in progress.

The date of the last search of all databases was 10 March 2021. Reference management software (EndNote version 20, Clarivate Analytics, Boston, MA, USA) was used to identify and remove potentially duplicated records. Two review authors (KK and SA) independently screened all potentially relevant records and classified the studies according to the criteria provided in the Cochrane Handbook for Systematic Reviews of Interventions [9]. Rayyan—a web and mobile application for systematic reviews (available at www.rayyan.ai)—was used for screening. All disagreements were resolved through discussion. We included all parallel-group randomized controlled trials (RCTs) and considered cluster RCTs for inclusion. Crossover studies that were not applicable and nonrandomized studies were excluded. Studies were included regardless of the publication status or language of publication.

### 2.2. Type of Participants

We defined the eligible patient population as all patients undergoing open or laparoscopic anterior resection and low anterior resection for curable colorectal cancer. Trials including patients with massive invasion of colorectal cancer into adjacent organs that could not be resected, synchronous unresectable metastasis or peritoneal metastasis, and those with inoperable disease owing to comorbidities were excluded.

### 2.3. Types of Interventions and Comparators

We compared HL and LL. Concomitant interventions had to be the same in the experimental and comparator groups to establish fair comparisons. HL was defined as IMA ligation immediately after it branches off the anterior surface of the abdominal aorta, whereas LL was defined as IMA ligation at the level of the superior rectal artery, preserving the LCA.

### 2.4. Type of Outcomes

We did not measure the outcomes assessed in this review as eligibility criteria.

### 2.5. Primary Outcomes

Overall mortality (OM) and overall postoperative complications were regarded as the primary outcome measures. OM was defined as the length of time from randomization to death from any cause. Overall postoperative complications were defined as complications occurring within 30 days after surgery that could be classified according to the Clavien–Dindo classification.

### 2.6. Secondary Outcomes

Disease recurrence (DR), cancer-specific mortality (CSM), postoperative mortality, anastomotic leakage, defecatory dysfunction, urinary dysfunction, and sexual dysfunction were regarded as secondary outcome measures. DR was defined as the length of time from randomization to recurrence. CSM was defined as the length of time from randomization to cancer-related death. Postoperative mortality was defined as the number of deaths within 30 days after surgery. Anastomotic leakage was defined as incontinuity at the anastomotic site detected clinically or radiologically within 30 days after surgery. Defecatory dysfunction was assessed using the Fecal Incontinence Quality of Life (FIQL) scale, Jorge–Wexner Incontinence Score (JWIS), Agachan–Wexner Constipation Score (AWCS), or the Gastrointestinal Quality of Life Index (GIQLI). Urinary dysfunction was assessed using the International Consultation on Incontinence Questionnaire—Urinary Incontinence (ICIQ-UI) and International Prostate Symptom Score (IPSS). Sexual dysfunction was assessed using the International Index of Erectile Function (IIEF) and the Female Sexual Function Index (FSFI).

We considered outcomes for defecatory, urinary, and sexual dysfunction measured up to and including 6 months after randomization as short-term, and those beyond 6 months as long-term outcomes.

### 2.7. Assessment of Risk of Bias in Included Studies

Two review authors (KK and SA) independently assessed the risk of bias in each included study. All disagreements were resolved through discussion. We planned to assess the risk of bias of the RCTs using the Cochrane risk of bias tool for randomized trials. The risk of bias domains were “low risk,” “high risk,” or “unclear risk,” and they were evaluated using individual items, as described in the Cochrane Handbook for Systematic Reviews of Interventions [10].

### 2.8. Data Collection and Analysis

Outcome data were extracted as needed to calculate summary statistics and measures of variance. The collected information for the included studies is provided in Table S4. For dichotomous outcomes, we obtained the number of events and their proportions, and the summary statistics with corresponding measures of variance. For continuous outcomes, we obtained the means, standard deviations, or other necessary data. We calculated the hazard ratios (HRs) using the method of Tierney et al. [11] and the corresponding 95% confidence intervals (CIs) for time-to-event outcomes, and analyzed the data using a random-effects model. Review Manager 5 software (The Cochrane Collaboration, Copenhagen, Denmark) was used for statistical analysis. We assessed the impact of heterogeneity on the meta-analysis and interpreted it according to the guidelines of the Cochrane Handbook for Systematic Reviews of Interventions [9]. We expected the characteristics, such as age (younger than 65 years versus older than 65 or 65 years of age), adjuvant therapy (adjuvant therapy versus no adjuvant therapy), and tumor stage (localized versus locally advanced versus advanced), to be heterogeneous and planned to carry out subgroup analyses with an investigation of the interactions limited to primary outcomes. Sensitivity analyses of primary outcomes were only planned for RCTs to explore the influence of the risk of bias (when applicable) on the effect sizes by excluding studies with high or unclear risks. However, we could not perform secondary analyses because there were no relevant data, and the RCTs were scarce. If there were at least 10 studies investigating a particular outcome, funnel plots were used to assess small-study effects.

### 2.9. Summary of Findings Table

We assessed the overall certainty of the evidence (CoE) for each outcome according to GRADE. Two review authors (KK and JHJ) independently rated the CoE for each outcome, and resolved any discrepancies by consensus. We considered the criteria related to internal validity (risk of bias, inconsistency, imprecision, and publication bias) and external validity, such as the directness of results [12].

## 3. Results

### 3.1. Search Results

**Figure 1:**
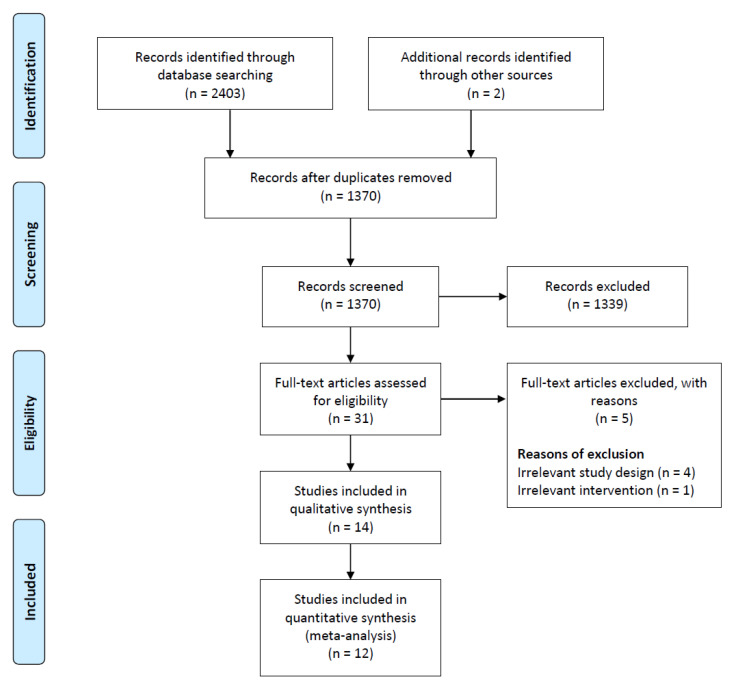
Prisma flow chart of this study.

In total, 2403 records were identified through our database searches. A gray literature repository was also found. Two additional records were identified [13,14]. After removing duplicates, initial screening was performed for the titles and abstracts of 1370 records, and 1339 records were excluded. After the initial screening, screening of the full text of 31 articles was performed, and 5 full-text articles were excluded for the following reasons: nonrandomized articles (4 articles) [15,16,17,18] and different interventions (1 article) [19]. Two studies (two articles) were ongoing [20,21]. Finally, 12 RCTs (24 articles) that met the inclusion criteria were included for qualitative synthesis in this review. The assessment process is illustrated in the Preferred Reporting Items for Systematic Reviews and Meta-Analyses flowchart (Figure 1).

### 3.2. Included Studies

Twelve published full-text studies were identified [14,22,23,24,25,26,27,28,29,30,31,32,33]. Two published full-text journal articles [29,30] reporting different outcomes were from one RCT. Eight studies were published in English and four studies in Chinese [22,24,32,33]. We translated the Chinese full-text articles with the use of professional translators. These RCTs were conducted in various countries, including China [22,24,25,28,32,33], Italy [23,26,27], Japan [29,30,31], and Poland [14]. We attempted to contact all corresponding authors and indicated contact persons to obtain additional information on the study methodology and results, and received replies for two studies (Table S5). Table 1 shows the baseline characteristics of the included studies. All studies were single-center studies, except for one [27], and were performed between 2008 and 2019.

A total of 1431 randomized participants were included in the studies. The mean age of the RCTs, participants ranged from 49.9 to 69.0 years. The total number of participants with stage 0/I disease was 445; stage II, 390; stage III, 400; and stage IV, 27. The number of participants by stage was not reported in two RCTs [24,28]. Five RCTs [14,23,25,26,33] excluded patients with stage IV disease, and two RCTs [22,32] did not report the number of patients with stage IV disease. Fiori et al. [23] conducted their study on patients with sigmoid colon cancer; Kruszewski et al. [14] included patients with rectosigmoid colon cancer; and all other studies were conducted among patients with rectal cancer. Participants who underwent only laparoscopic surgery were recruited in eight RCTs [22,23,25,26,27,28,32,33], whereas other RCTs recruited participants who underwent laparoscopic or open surgery [14,24,29,30,31]. Patients who underwent neoadjuvant chemoradiotherapy or radiotherapy were excluded from eight RCTs [22,24,25,26,28,31,32,33]. A total of 294 patients (HL: 149 and LL: 135) underwent adjuvant chemotherapy, and 181 participants (HL: 86 and LL: 95) underwent protective stoma formation (Table 1).

**Figure 2:**
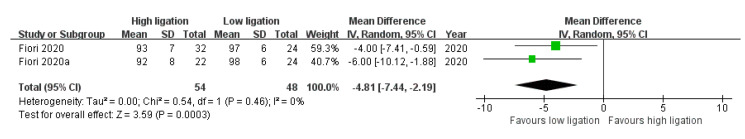
Forest plot of defecatory dysfunction assessed by the overall score of the fecal incontinence quality of life scale (short-term).

**Figure 3:**
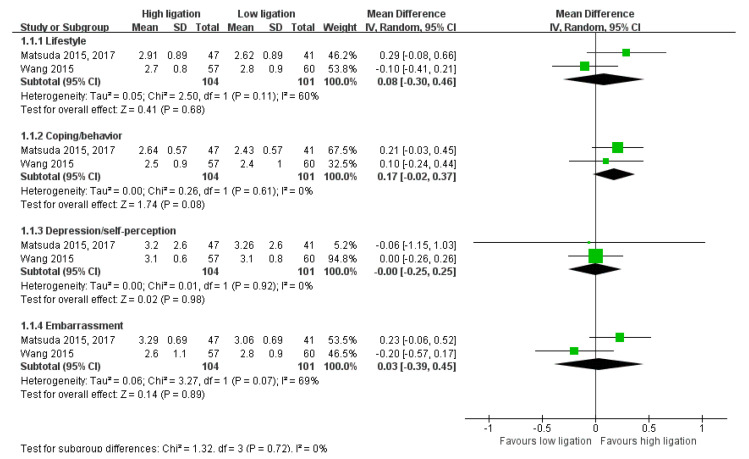
Forest plot of defecatory dysfunction assessed by each domain of the fecal incontinence quality of life scale (short-term).

**Figure 4:**
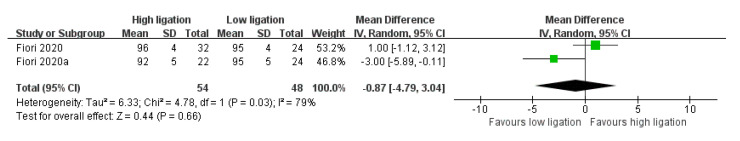
Forest plot of defecatory dysfunction assessed by the overall score of the fecal incontinence quality of life scale (long-term).

**Figure 5:**
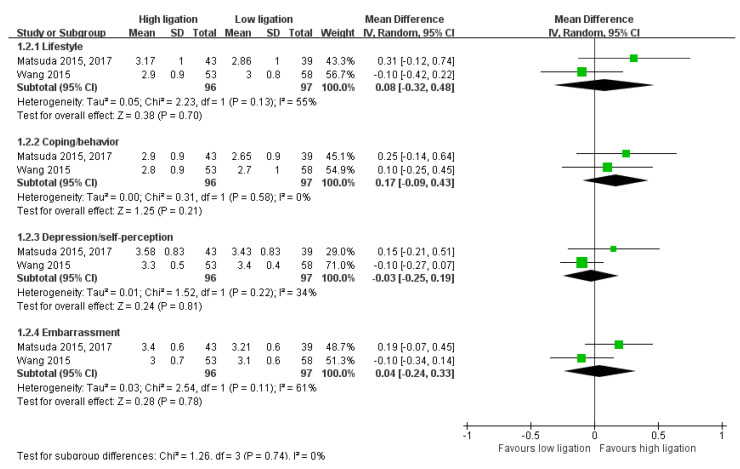
Forest plot of defecatory dysfunction assessed by each domain of the fecal incontinence quality of life scale (long-term).

The OM rate was available in four studies [14,25,29,31]. The 2-year OM rate was available in one study [25]. The OM of patients with locally advanced disease was available in two studies [29,31]. DR was reported in six studies [14,22,24,25,29,31]. The DR of patients with locally advanced disease was available in three studies [14,29,31]. Postoperative complications were reported in 10 studies [14,23,24,25,26,27,30,31,32,33]. CSM was available in only one study [14]. Postoperative mortality was reported in eight studies [14,23,25,26,27,30,31,32]. Unpublished data regarding postoperative mortality were obtained by e-mail from the author of one study [25]. Anastomotic leakage was reported in 12 studies [14,22,23,24,25,26,27,28,30,31,32,33]. Four studies reported defecatory dysfunction using the FIQL scale [23,24,26,30]. These four studies used different versions of the FIQL scale [34,35,36]. Two studies reported the FIQL as the overall score [23,26], while others reported it separately for each domain [24,30]. However, there were differences in the FIQL scales between those studies; therefore, we could not pool the effect estimates and assess the CoE (Figure 2, Figure 3, Figure 4 and Figure 5). Defecatory dysfunction was assessed by the JWIS in four studies [23,24,26,30]. Two studies reported defecatory dysfunction assessed by the AWCS [23,26]. One study reported defecatory dysfunction using the GIQLI [27]. Sexual dysfunction was reported in two studies by validated questionnaire (male: IIEF and female: FSFI) [26,27]. Urinary dysfunction measured by the ICIQ-IU or IPSS was reported in two studies [26,27].

Guo et al. [28] was funded by the Health Project of Jilin Province, China, and Zhou et al. [32] was funded by the Guangzhou Important Special Program of Health Medicine Cooperation and Innovation (Grant number: 201604020005). All authors of the included studies declared no conflicts of interest.

### 3.3. Excluded Studies

We excluded 4 studies [15,16,17,19] (5 articles) of 18 studies (29 articles) after the evaluation of the full-text articles. Three studies (four articles) were not RCTs: two were nonrandomized studies [16,17] and one was a retrospective study [15] using RCT data collected for other purposes. The intervention and comparator in one excluded study were IMA dissection first versus inferior mesenteric vein dissection, which were different from the intervention and comparator in our study [19] (Table S6).

### 3.4. Risk of Bias of Included Studies

**Figure 6:**
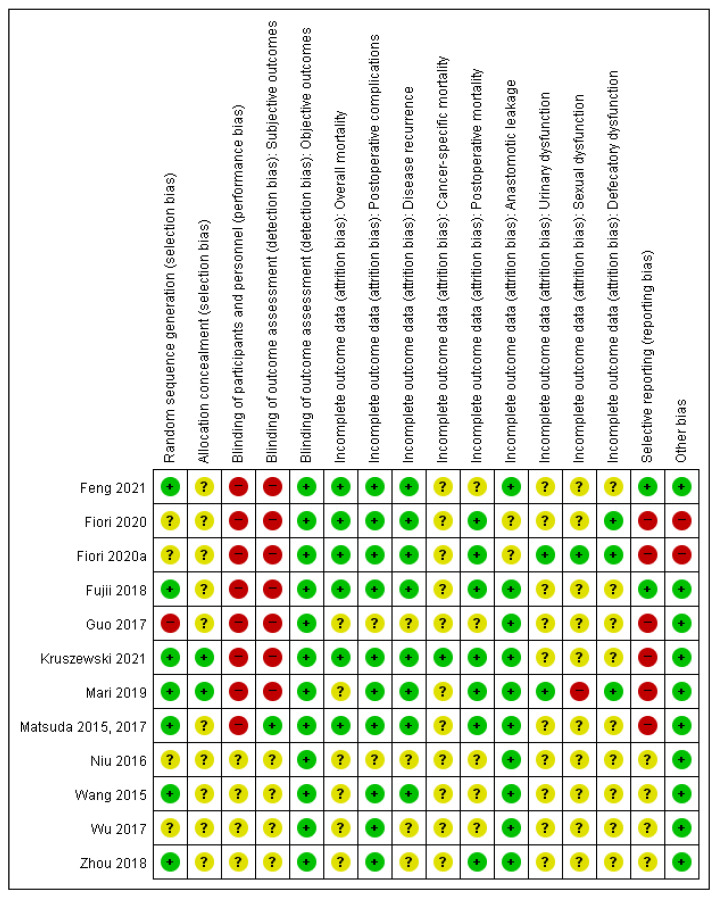
Risk of bias summary: Review authors’ judgments about each risk of bias item for each included study. Subjective outcomes: Postoperative complications, anastomotic leakage, urinary dysfunction, sexual dysfunction, and defecatory dysfunction. Objective outcomes: Overall mortality, disease recurrence, cancer-specific mortality, and postoperative mortality. Categories: Green point (+) = low risk of bias; yellow point (?) = unclear risk of bias; red point (−) = high risk of bias.

The details of the risk of bias in the included studies are described in Figure 6.

Seven studies were classified as having a low risk of bias for random sequence generation [14,24,25,27,29,30,31,32]. Two studies were classified as having a low risk of bias for allocation concealment [14,27]. Eight studies were rated as having a high risk of bias for blinding participants and personnel because double blinding was impossible owing to the surgical trial nature [14,23,25,26,27,28,29,30,31]. Other studies were rated as having an unclear risk of bias owing to the lack of information regarding the blinding method [22,24,32,33]. One RCT was classified as having a low risk of bias for blinding of the outcome assessment of subjective outcomes (postoperative complications, anastomotic leakage, defecatory dysfunction, urinary dysfunction, and sexual dysfunction), because the investigators and outcome assessors were blinded in these studies [29,30]. Six studies were classified as having a high risk of bias for selective reporting because the outcomes in the material and methods section were not the same as the actual reported outcomes [14,23,26,27,28,29,30]. Two studies were classified as having a high risk of bias for other biases because the protocol of the studies was different from that of the published journal [23,26].

### 3.5. Effects of Interventions

#### 3.5.1. Primary Outcomes

1. OM; four RCTs with 649 participants (HL: 327 and LL: 322) were analyzed for OM [14,25,29,31]. We are very uncertain about the effects of HL in reducing OM (HR: 1.24, 95% CI: 0.85–1.83; I2 = 0%; very low CoE) (Table 2). We downgraded the CoE due to serious study limitations and very serious imprecision;
2. Postoperative complications; ten RCTs with 1293 participants (HL: 657 and LL: 636) were analyzed for postoperative complications [14,23,24,25,26,27,30,31,32,33]. There may be little to no difference in the postoperative complications between HL and LL (risk ratio (RR): 1.15, 95% CI: 0.87–1.52; I2 = 44%; low CoE) (Table 2). We downgraded the CoE due to serious study limitations and serious inconsistencies.

#### 3.5.2. Secondary Outcomes

1. DR; analysis of 862 (HL: 436 and LL: 426) participants from six RCTs [14,22,24,25,29,31] showed a very uncertain effect of HL on reducing DR (HR: 1.17, 95% CI: 0.83–1.63; I2 = 0%; very low CoE) (Table 2). We downgraded the CoE due to serious study limitations and very serious imprecision;
2. CSM; analysis of 118 (HL: 59 and LL: 59) participants from one RCT [14] demonstrated a very uncertain effect of HL in reducing CSM (HR: 3.03, 95% CI: 1.18–7.77; very low CoE) (Table 2). We downgraded the CoE due to serious study limitations and very serious imprecision;
3. Postoperative mortality; analysis of 1051 (HL: 534 and LL: 517) participants from eight RCTs [14,23,25,26,27,30,31,32] was performed. We are very uncertain about the effect of HL on postoperative mortality (RR: 0.33, 95% CI: 0.03–3.14; I2 = 0%; very low CoE) (Table 2). We downgraded the CoE due to serious study limitations and very serious imprecision;
4. Anastomotic leakage; analysis of 1429 (HL: 721 and LL: 708) participants from 12 RCTs [14,22,23,24,25,26,27,28,30,31,32,33] was performed. We are very uncertain about the effects of HL on anastomotic leakage (RR: 1.32, 95% CI: 0.92–1.88; I2 = 0%; very low CoE) (Table 2). We downgraded the CoE due to serious study limitations, serious imprecision, and publication bias.
5. Functional Outcomes (Short-Term Follow-Up)

(1) Defecatory dysfunction

Analyses of 307 (HL: 158 and LL: 149) participants from four RCTs [23,24,26,30] for the JWIS, 102 (HL: 54 and LL: 48) participants from two RCTs [23,26] for the AWCS, and 196 (HL: 101 and LL: 95) participants from one RCT [27] for the GIQLI were performed.

HL may reduce defecatory function (constipation) assessed with the AWCS (mean difference (MD): 1.63, 95% CI: 0.85–2.42; I2 = 64%; low CoE). There may be little to no difference in defecatory dysfunction assessed with the JWIS (MD: 0.42, 95% CI: 0.20–0.63; I2 = 0%; low certainty of evidence) and with the GIQLI (MD: −1.13, 95% CI: −3.32 to 1.06; low CoE). We downgraded the CoE of each questionnaire due to serious imprecision and serious study limitations (Table 3).

(2) Urinary dysfunction

Two hundred and forty-two (HL: 123 and LL: 119) participants from two RCTs [26,27] were analyzed for the ICIQ-UI. Analysis of 196 (HL: 101 and LL: 95) participants from one RCT [27] for the IPSS was performed. There may be little to no difference in urinary dysfunction assessed with the ICIQ-UI (MD: 1.44, 95% CI: 0.70–2.17; I2 = 0%; low CoE) and the IPSS between HL and LL (MD: 1.69, 95% CI: −0.27 to 3.65; low CoE) (Table 3). We downgraded the CoE of each questionnaire due to serious imprecision and serious study limitations.

(3) Sexual dysfunction

Analyses of 158 (HL: 84 and LL: 74) participants from two RCTs [26,27] for IIEF-5 and 46 (HL: 22 and LL: 24) participants from one RCT [26] for FSFI were performed. There may be little to no difference in male sexual dysfunction assessed with the IIEF-5 (MD: −3.73, 95% CI: −5.46 to −2.01; I2 = 0%; low CoE). Female sexual function assessed with the FSFI was significantly decreased in HL compared with LL (MD: −5.00, 95% CI: −7.03 to −2.97; low CoE) (Table 3) We downgraded the CoE of each questionnaire due to serious imprecision and serious study limitations.

6. Functional Outcomes (Long-Term Follow-Up)

(1) Defecatory dysfunction

Two hundred and ninety-five (HL: 150 and LL: 145) participants from four RCTs [23,24,26,30] for the JWIS, 102 (HL: 54 and LL: 48) participants from two RCTs [23,26] for the AWCS, and 196 (HL: 101 and LL: 95) participants from one RCT [27] for the GIQLI were analyzed.

HL may reduce defecatory function assessed with the AWCS (MD: 1.61, 95% CI: 0.83–2.39; I2 = 70%; low CoE). There may be little to no difference in defecatory dysfunction assessed with the JWIS (MD: 0.11, 95% CI: −0.25 to 0.47; I2 = 61%; low CoE) and the GIQLI (MD: −4.30, 95% CI: −6.34 to −2.26; low CoE) (Table 4). We downgraded the CoE for each questionnaire due to serious imprecision and serious study limitations.

(2) Urinary dysfunction

Two hundred and forty-two (HL: 123 and LL: 119) participants from two RCTs [26,27] for the ICIQ-UI were analyzed. There may be little to no difference in urinary dysfunction assessed with the ICIQ-UI between HL and LL (MD: 1.90, 95% CI: 0.82–2.99; I2 = 54%; low CoE) (Table 4). This was downgraded due to serious imprecision and serious study limitations. Analysis of 196 (HL: 101 and LL: 95) participants from one RCT [27] for the IPSS was performed. HL may aggravate urinary symptoms assessed with the IPSS compared with LL (MD: 4.72, 95% CI: 2.43–7.01; low CoE) (Table 4). This was downgraded due to serious imprecision and serious study limitation.

(3) Sexual dysfunction

Analyses of 158 (HL: 84 and LL: 74) participants from two RCTs [26,27] for the IIEF-5 and 46 participants (HL: 22 and LL: 24) for the FSFI were performed. HL may reduce sexual function assessed with the IIEF-5 (MD: −5.11, 95% CI: −6.85 to −3.37; I2 = 0%; low CoE) and the FSFI (MD: −5.00, 95% CI: −6.74 to −3.26; low CoE) compared with LL (Table 4). We downgraded the CoE for each questionnaire due to serious imprecision and serious study limitations.

### 3.6. Subgroup Analysis

**Figure 7:**
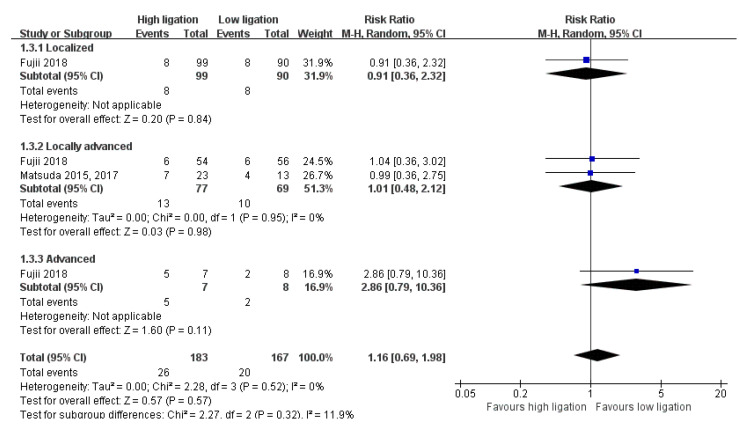
Forest plot of the subgroup analysis for 5-year overall mortality.

**Figure 8:**
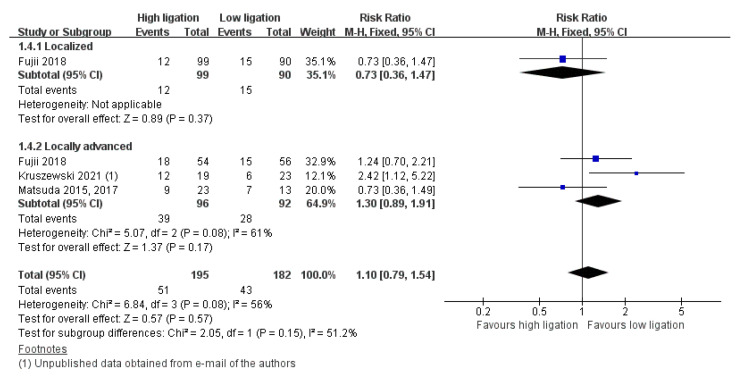
Forest plot of the subgroup analysis for 5-year disease recurrence.

In terms of OM, we found an RR of 0.91 (95% CI 0.36–2.32) with localized disease versus an RR of 1.01 (95% CI 0.48–2.12) with locally advanced disease versus an RR of 2.86 (95% CI 0.79–10. 36) with advanced disease. The test for interaction showed no evidence of a difference between the subgroups *(p* = 0.32, I2 = 11.9 %) (Figure 7). In terms of DR, we found an RR of 0.73 (95% CI 0.36–1.47) with localized disease versus an RR of 1.30 (95% CI 0.89–1.91) with locally advanced disease. The test for interaction showed no evidence of a difference between the subgroups (*p* = 0.15, I2 = 51.2 %) (Figure 8). Other subgroup analyses were not performed because of the limited data.

## 4. Discussion

Our study showed a very uncertain effect of HL on improving survival outcomes compared with LL. Several studies have demonstrated that apical lymph node metastasis is a prognostic factor for overall survival (OS) and disease-free survival (DFS) [42,43,44]. In general, HL is considered to be oncologically safer than LL because more lymph nodes, including apical lymph nodes, can be removed during HL. However, although our study showed a very uncertain effect of HL on improving survival outcomes, HR was higher in HL than LL. What should be considered is whether apical lymph node dissection (ALND) was performed during LL and whether ALND was performed appropriately during HL in each RCT. LL of the IMA with ALND was performed in six studies among all of the enrolled studies. Other studies did not report whether ALND was performed during LL. In fact, the Japanese guidelines recommend that D3 lymph node dissection can be performed for T2 or more advanced diseases [45]. Many centers perform LL with ALND on patients with clinically suspected apical lymph node metastasis [46]. Therefore, although seven studies did not report whether ALND was performed, we speculate that ALND was likely performed when apical lymph node metastasis was clinically suspected. Although a single study cannot represent all enrolled studies, Fujii et al. [31] reported that the number of harvested lymph nodes and metastatic lymph nodes around the IMA root was not different between HL and LL. Thus, lymphadenectomy during LL may be performed appropriately in enrolled RCTs. In contrast, the higher HR of HL may suggest the possibility of inappropriate ALND during HL. ALND may be incompletely performed to avoid autonomic nerve injury during HL. In fact, Turgeon et al. [47] reported that the proportion of patients with a number of harvested lymph nodes of < 12 was larger in HL than LL. For the more accurate evaluation of survival outcomes, more rigorous trials in which standardized HL, LL, and the same extent of lymphadenectomy during HL are performed are necessary. 

This study showed that HL may not increase postoperative complications. In addition, the effect of HL on postoperative mortality was very uncertain because only two patients died in the LL group. These findings suggest that the level of IMA ligation does not lead to a difference in the postoperative complications and mortality.

A major complication of colon resection for sigmoid and rectal cancers is anastomotic leakage. Our study showed a very uncertain effect of HL on anastomotic leakage. Proponents of LL believe that performing LL maintains a better blood supply to the proximal colonic limb. Komen et al. [16] reported that the blood flow at the proximal colonic limb increased after LL, whereas it was not significantly decreased after HL. Guo et al. [28] reported that the marginal artery stump pressure was significantly higher in patients who underwent LL than in patients who underwent HL. Han et al. [48] reported that the time of perfusion to the colon could be more delayed after HL, but the total intensity of perfusion was similar between HL and LL in perfusion tests using intraoperative indocyanine green angiography. However, none of these studies reported a significant difference in the incidence of anastomotic leakage between HL and LL. These results indicated that, although the blood flow to the proximal colonic limb may be lower after HL than after LL, the relatively low blood flow after the HL of the IMA may be sufficient for anastomotic healing.

Another factor affecting anastomotic healing after colorectal surgery is tension between the proximal and distal colonic limbs. HL may allow tension-free anastomosis to be achieved. Some studies have reported that a much longer colonic length could be gained after HL than after LL [49,50,51]. Therefore, the lack of a difference in the incidence of anastomotic leakage between HL and LL in this study may be explained by the sufficient blood flow to the proximal colonic limb after HL and the ease of tension-free anastomosis.

Interestingly, a large, multi-institutional study with 877 patients conducted in the US showed that LL was not inferior compared with HL in terms of the anastomotic leak rate, locoregional recurrence, DFS, and OS, which is similar to the results of our study [47].

Our study showed that HL may aggravate constipation based on the minimal clinically important difference (MCID). Injury to the superior hypogastric plexus (SHP) around the IMA is common during HL, and nerve injuries may lead to defecatory dysfunction. Denervation of the proximal anastomotic site may lead to colonic hypomotility, inefficient intestinal content transport, and upstream colonic gas retention [23,30]. Long denervation of the SHP during HL may lead to a severe change in the proximal colon compared with that during LL, and this change may lead to feelings of incomplete evacuation and abdominal pain on the left side.

The inferior hypogastric plexus (IHP) is interconnected with SHP via the inferior mesenteric ganglia acting as junctions. The IHP receives pelvic parasympathetic fibers from roots S2–S5 (splanchnic nerves). These nerves are covered by the parietal fascia, pierce the endopelvic fascia, cross the retrorectal space, and form branches into the rectum via the lateral ligaments [52]. Nerve fibers from the IHP innervate the seminal vesicle, prostate, bladder, cervix, and vagina. The IHP is also responsible for penile erection, ejaculation, detrusor contractility, female arousal, and vaginal lubrication. Urinary function depends on the parasympathetic nerves for bladder emptying and the sympathetic nerves for urinary continence. Incontinence and urgency may occur if the IHP is injured. Male sexual function requires the coordination of parasympathetic nerves for erection and sympathetic nerves for ejaculation, while both nerves play a similar role in sexual arousal in females. Thus, injury to these nerves leads to erectile dysfunction and retrograde ejaculation in males, and dyspareunia in females. Injury to the SHP and inferior mesenteric ganglia may lead to the same urinary and sexual dysfunction occurring in cases of injury to the IHP due to interconnectivity. Significant urinary dysfunction assessed by the IPSS, male sexual dysfunction assessed by the IIEF-5, and female sexual dysfunction assessed by the FSFI were reported as long-term outcomes (beyond 6 months), whereas only significant female sexual dysfunction was reported within the 6 months after surgery based on the MCID. Nerve injury can be permanent due to complete nerve fiber transection or can be reversible when stretching or compression occurs [53]. Complete nerve transection may be avoided and permanent urinary and sexual dysfunction may be prevented when LL is performed instead of HL. Late improvement after partial nerve injury may have occurred in the LL group.

HL and LL in colorectal cancer surgery have been investigated in recent reviews [4,13,46,54,55]. Similar to our results, Hajibandeh et al. [13] reported no significant differences in anastomotic leakage, postoperative complications, postoperative mortality, OS, and DFS, and Kong et al. [4] found no significant difference in anastomotic leakage between HL and LL. In contrast, Jonnada et al. [46] reviewed 31 studies and reported that the LL of the IMA was associated with decreased rates of colorectal anastomotic leaks, urinary dysfunction, and overall postoperative morbidity. This study analyzed functional outcomes, including urinary dysfunction, as a dichotomous variable. However, 24 studies among the 31 enrolled studies were nonrandomized studies, and urinary dysfunction was assessed in nonrandomized studies. In addition, only seven RCTs were included in the analysis of the anastomotic leakage. In particular, Yin et al. [54] used LL with high dissection of lymph nodes as an experimental intervention to compare with HL. This study reviewed four RCTs [22,27,28,31] and 13 non-randomized studies, and reported no significant differences in OS, DFS, and systemic recurrence between HL and LL with high dissection of lymph nodes. These results are similar to our results. In addition, this study reported that the LL of the IMA with high dissection of lymph nodes was associated with decreased rates of colorectal anastomotic leaks. Tryliskyy et al. [55] utilized two RCTs [26,27] to assess the genito-urinary function at 9 months following surgery using ICIQ-UI and IIEF, and reported that the LL of the IMA demonstrated significantly better ICIQ-UI and IIEF than HL. We included the same RCTs for long-term genito-urinary function in our study. Our statistical methods for long-term genito-urinary function differed from those used in the study. We adopted the mean difference for ICIQ-UI, since both RCTs employed the same questionnaire (ICIQ-UI short form). Using the method described by Thorlund et al. [56], we converted IIEF to IIEF-5, the more well-known questionnaire. They did not follow rigorous methodologies, such as the Methodological Expectations of Cochrane Intervention Reviews (MECIR), as laid out by the Cochrane Collaboration. One study reported the CoE according to the GRADE [4]. However, the MCID or the reasons for downgrading were not presented. One study reported functional outcomes using questionnaires [55]. However, it was limited to the genito-urinary function using ICIQ-UI and IIEF.

The first advantage of our study is that we followed rigorous Cochrane methodologies and the GRADE approach to assess CoE. Secondly, to the best of our knowledge, our study is the first systematic review to investigate important patient outcomes, including all assessable functional outcomes, using questionnaires. Finally, our study comprehensively reported oncologic outcomes, including CSM, not reported in previous systematic reviews.

The first limitation of our study was that we calculated hazard ratios according to raw data from original studies. However, it was inevitably performed for meta-analysis, because a heterogeneity of the unit of measurement was observed in the enrolled RCTs. Secondly, despite a comprehensive search strategy without any publication or language restrictions, we found only a small number of studies. In this review, these studies were insufficient to generate funnel plots; therefore, the risk of publication bias may have been underestimated. Thirdly, most of the enrolled RCTs had study limitations, because RCTs for surgical interventions cannot be fully blinded. Therefore, the CoE ranged from low to very low according to GRADE, which meant that the true effect may be substantially different from what the review showed. Therefore, additional studies of better quality comparing HL to LL appear to be essential. Future trials should be conducted with higher methodologic standards. Fourthly, few RCTs have reported the functional outcomes assessed using the questionnaires. In particular, discrepancies in the reporting method were observed in four RCTs that reported the result of the FIQL. Fifthly, although HL and LL were performed in the enrolled RCTs, HL and LL may not be standardized. In particular, some of the enrolled RCTs did not report whether ALND was performed; thus, clarifying whether ALND during HL was performed appropriately is necessary. Finally, the enrolled RCTs provided limited data regarding the use of neoadjuvant therapy and the number of participants who underwent laparoscopic surgery, adjuvant therapy, disease stage, protective stoma use, and ALND. Therefore, limited subgroup analyses based on these parameters were performed.

## 5. Conclusions

We are very uncertain about the effect of HL on OM, DR, and CSM. Postoperative complications may not be different between HL and LL. We are very uncertain about the effect of HL in terms of postoperative mortality and anastomotic leakage. In addition, LL may be beneficial for short-term and long-term defecatory dysfunction (constipation). LL may be more beneficial for long-term urinary symptoms and sexual function compared with HL. However, these results were based on the low CoE; therefore, more rigorous RCTs are necessary to evaluate the effects of HL and LL on the treatment of colorectal cancer.

## Figures and Tables

**Table 1:** (**a**) Baseline characteristics of included studies. (**b**) Baseline characteristics of included studies.

(**a**)																					
**Study Name**	**Trial Period (Year to Year)**	**Study Design/Setting/Country**	**Description of Participants**									**Intervention and Comparator**		**Duration of Follow-Up (Months)**			**Total Number Analyzed**			**Age (Mean ± Standard Deviation)**	
Feng 2021 [25]	2016 to 2018	RCT/likely single-center/China	Patients 18–75 years old with histologically proven adenocarcinoma, a rectal lesion (distal margin 5–15 cm from the anus), clinical stage I–III disease (based on CT or MRI), and a Karnofsky score of ≥80 (unable to perform strenuous physical activity but ambulatory and able to perform light or sedentary work)									HL		24			47			60.5 ± 10.2	
												LL					48			59.8 ± 8.9	
Fiori 2020 [23]	2013 to 2018	RCT/single-center/Italy	Patients with stage II, stage III, M0, and sigmoidal cancer treated by laparoscopic surgery									HL		60			32			67.0 ± 9.0	
												LL					24			68.0 ± 10.0	
Fiori 2020a [26]	2013 to 2019	RCT/single-center/Italy	Patients treated with curative laparoscopic resection for pT2N0M0, rectal adenocarcinoma, and laparoscopic TME									HL		60			22			68.0 ± 9.0	
												LL					24			68.0 ± 11.0	
Fujii 2018 [31]	2006 to 2012	RCT/single-center/Japan	Patients aged 20 years or above, with histologically proven adenocarcinoma of the rectum.									HL		60			164			65.9 ± 10.4	
												LL					160			65.6 ± 11.5	
Guo 2017 [28]	2013 to 2013	RCT/single-center/China	Patients with only solitary radical resectable rectal cancers 3–20 cm from the anus as their first malignant neoplasm									HL		NR			29			NR	
												LL					28			NR	
Kruszewski 2021 [14]	2010 to 2016	RCT/single-center/Poland	Patients who underwent radical surgery due to rectal or rectosigmoid adenocarcinoma									HL		More than 60 months			65			64.0 ± 9.0	
												LL					65			65.0 ± 8.5	
Mari 2019 [27]	2014 to 2016	RCT/multi-center/Italy	Patient 18 years of age or older, BMI < 30, ASA I, II, III, Elective laparoscopic LAR + TME, and no evidence of metastatic disease									HL		12			101			67.0 (34.0–87.0) ^a,b^	
												LL					95			68.0 (35.0–86.0) ^a,b^	
Matsuda 2015 [30] ^c^	2008 to 2011	RCT/single-center/Japan	Patients scheduled for anterior resection with reconstruction using the double-stapling technique for rectal cancer									HL		12			51			69.0 (45.0–85.0) ^d^	
												LL					49			67.0 (45.0–89.0) ^d^	
Matsuda 2017 [29] ^c^	2008 to 2011	RCT/single-center/Japan	Patients with curable rectal cancer located <15 cm from the anus and patients with end-to-end anastomosis reconstructed by the double-stapling technique									HL		36			51			69.0 ^d^	
												LL					49			67.0 ^d^	
Niu 2016 [22]	2009 to 2015	RCT/single-center/China	All patients with rectal cancer confirmed by preoperative colonoscopic pathology									HL		NR			45			49.9 ± 8.2	
												LL					54			51.3 ± 6.3	
Wang 2015 [24]	2013 to 2013	RCT/single-center/China	Patients with rectal cancer undergoing low anterior resection, R0 resection, and end-to-end double anastomosis									HL		12			63			56.8 ± 14.2	
												LL					65			58.6 ± 13.7	
Wu 2017 [33]	2014 to 2016	RCT/single-center/China	Patients with low rectal cancer without invasion or adhesion to other organs or structures; patients under the age of 70 years and able to tolerate laparoscopic surgery; patients without severe cardiopulmonary disease, renal dysfunction, dyshepatia, or metabolic disorders; without metastasis; without intestinal obstruction, perforation, or gastroenteritis; and with no history of radiotherapy or chemotherapy									HL		NR			50			58.4 ± 9.3	
												LL					46			59.1 ± 9.1	
Zhou 2018 [32]	2015 to 2016	RCT/single-center/China	Patients with rectal cancer who were confirmed to have complete resection of the primary tumor and no distant metastasis, 2 to 15 cm from the anus, after preoperative examination; patients aged 18 to 75 years old who could undergo laparoscopic surgery and who had no obvious contraindications to surgery									HL		1			52			52.7 ± 12.9	
												LL					52			53.9 ± 13.5	
(**b**)																					
**Study**	**Procedure**		**Tumor Location**	**Stage**								**Neoadjuvant CRT**			**Adjuvant CTx**			**Protective Stoma**			**ALND for LL**
				**0/I**		**II**		**III**		**IV**		**HL**	**LL**		**HL**	**LL**		**HL**	**LL**		
				**HL**	**LL**	**HL**	**LL**	**HL**	**LL**	**HL**	**LL**										
Feng 2021 [25]	Laparoscopic LAR		Rectum	21 ^a^	25 ^a^	12 ^a^	13 ^a^	14 ^a^	10 ^a^	Excluded		Excluded			NR			NR			Yes
Fiori 2020 [23]	Laparoscopic anterior rectosigmoid resection		Sigmoid	Excluded		10 ^a^	8 ^a^	22 ^a^	16 ^a^	Excluded		NR			NR			NR			NR
Fiori 2020a [26]	Laparoscopic AR		Rectum	22 ^b^	24 ^b^	Excluded		Excluded		Excluded		Excluded			NR			NR			NR
Fujii 2018 [31]	Laparoscopic or open AR		Rectum	60 ^b^	60 ^b^	43 ^b^	36 ^b^	54 ^b^	56 ^b^	7 ^b^	8 ^b^	Excluded			39 ^b^	46 ^b^		36 ^b^	47 ^b^		Yes
Guo 2017 [28]	Laparoscopic resection		Rectum	NR								Excluded ^c^			NR			10 ^b^	10 ^b^		Yes
Kruszewski 2021 [14]	Laparoscopic or open, AR or HP or APR		Rectum or rectosigmoid	32 ^a^	23 ^a^	14 ^a^	18 ^a^	19 ^a^	24 ^a^	Excluded		42 ^a^	43 ^a^		25 ^b^	27 ^b^		3 ^b^	2 ^b^		No
Mari 2019 [27] ^d^	Laparoscopic LAR		Rectum	44 ^e^	60 ^e^	25 ^a^	21 ^a^	39 ^e^	19 ^e^	3 ^a^	3 ^a^	30 ^a^	25 ^a^		56 ^a^	42 ^a^		NR			Yes
Matsuda 2015 [30] ^f^	Laparoscopic or open AR		Rectum	9 ^e^	17 ^e^	15 ^e^	17 ^e^	23 ^e^	13 ^e^	4 ^e^	2 ^e^	2 ^b^	5 ^b^		NR			20 ^b^	19 ^b^		NR
Matsuda 2017 [29] ^f^	Laparoscopic or open AR		Rectum	9 ^e^	17 ^e^	15 ^e^	17 ^e^	23 ^e^	13 ^e^	4 ^e^	2 ^e^	2 ^a^	5 ^a^		29 ^a^	20 ^a^		NR			NR
Niu 2016 [22]	Laparoscopic AR		Rectum	14 ^a^	19 ^a^	22 ^a^	25 ^a^	9 ^a^	8 ^a^	NR		Excluded			NR			4 ^e^	0 ^e^		Yes
Wang 2015 [24]	Laparoscopic or open LAR		Rectum	NR								Excluded ^c^			Excluded			Excluded			Yes
Wu 2017 [33]	Laparoscopic resection		Rectum	5 ^a^	4 ^a^	32 ^a^	29 ^a^	13 ^a^	13 ^a^	Excluded		Excluded			NR			NR			NR
Zhou 2018 [32]	Laparoscopic resection		Rectum	2 ^a^	4 ^a^	27 ^a^	23 ^a^	23 ^a^	25 ^a^	NR		Likely excluded ^c^			NR			13 ^a^	17 ^a^		NR

(**a**) HL, high ligation, LL, low ligation, NR, not reported, RCT, randomized controlled trial; ^a^. not defined; ^b^. mean age was based on randomized patients; ^c^. two articles were from 1 RCT; ^d^. median (range). (**b**) ALND, apical lymph node dissection, AR, anterior resection, CRT, chemoradiotherapy, CTx, chemotherapy, HL, high ligation, HO, Hartmann’s procedure, LAR, low anterior resection, LL, low ligation, NR, not reported; ^a^. no significant difference; ^b^. *p*-value not reported; ^c^. preoperative radiotherapy was excluded; ^d^. Baseline characteristics of Mari et al. [27] were based on randomized patients (*n* = 214); ^e^. statistically significant; ^f^. Two articles were from 1 RCT.

**Table 2:** Summary of findings: high ligation compared with low ligation in colorectal cancer surgery (primary and secondary outcomes, except for functional outcomes).

Patient or population: Colorectal cancer surgery Setting: Randomized controlled trials Intervention: High ligation Comparison: Low ligation						
Outcomes	Number of Participants (Studies)	Certainty of the Evidence (GRADE)	Relative Effect (95% CI)	Anticipated Absolute Effects		What Happened?
				Risk with Low Ligation	Risk Difference with High Ligation	
Overall mortality Follow-up: range 2 years to 5 years MCID: 2% absolute difference	649 (4 RCTs)	⨁◯◯◯ VERY LOW ^a^^,b^	**HR: 1.24** (0.85 to 1.83)	146 per 1000	**32 more per 1000** (20 fewer to 105 more)	We are very uncertain about the effects of HL on improving overall mortality
Postoperative complications Follow-up: 30 days MCID: 5% absolute difference	1293 (10 RCTs)	⨁⨁◯◯ LOW ^a^^,c,d^	**RR: 1.15** (0.87 to 1.52)	280 per 1000	**42 more per 1000** (36 fewer to 146 more)	There may be little to no difference in postoperative complications between HL and LL
Disease recurrence Follow-up: range 1 year to 5 years MCID: 2% absolute difference	862 (6 RCTs)	⨁◯◯◯ VERY LOW ^a^^,b^	**HR: 1.17** (0.83 to 1.63)	146 per 1000	**23 more per 1000** (23 fewer to 81 more)	We are very uncertain about the effects of HL on improving disease recurrence
Cancer-specific mortality Follow-up: 5 years MCID: 2% absolute difference	118 (1 RCT)	⨁◯◯◯ VERY LOW ^a^^,f^	**HR: 3.03** (1.18 to 7.77)	102 per 1000	**176 more per 1000** (17 more to 464 more)	We are very uncertain about the effects of HL on improving cancer-specific mortality
Postoperative mortality Follow-up: 30 days MCID: 2% absolute difference	1051 (8 RCTs)	⨁◯◯◯ VERY LOW ^a^^,f^	**RR: 0.33** (0.03 to 3.14)	4 per 1000	**3 fewer per 1000** (4 fewer to 8 more)	We are very uncertain about the effects of HL on improving postoperative mortality
Anastomotic leakage Follow-up: 30 days MCID: 5% absolute difference	1429 (12 RCTs)	⨁◯◯◯ VERY LOW ^a^^,e,g^	**RR: 1.32** (0.92 to 1.88)	65 per 1000	**21 more per 1000** (5 fewer to 57 more)	We are very uncertain about the effects of HL on improving anastomotic leakage
**The risk in the intervention group** (and its 95% CI) is based on the assumed risk in the comparison group and the **relative effect** of the intervention (and its 95% CI). **CI:** confidence interval; **MCID:** minimal clinically important difference; **RCT:** randomized controlled trial; **HR:** hazard ratio; **HL:** high ligation; **LL:** low ligation; **RR:** risk ratio						
**GRADE working group grades of evidence** **High certainty:** We are very confident that the true effect lies close to that of the estimate of the effect **Moderate certainty:** We are moderately confident in the effect estimate: The true effect is likely to be close to the estimate of the effect, but there is a possibility that it is substantially different **Low certainty:** Our confidence in the effect estimate is limited: The true effect may be substantially different from the estimate of the effect **Very low certainty:** We have very little confidence in the effect estimate: The true effect is likely to be substantially different from the estimate of the effect						

^a^. Downgraded by one level due to study limitations: allocation was clearly not concealed in most of the included studies, and/or participants were clearly not blinded in the included studies; ^b^. Downgraded by two levels due to imprecision: wide confidence interval crosses the assumed threshold of clinically important difference; ^c^. Downgraded by one level due to inconsistency due to clinically important heterogeneity; ^d^. Not downgraded further due to imprecision: wide confidence intervals attributed to the observed inconsistency (for which we rated down); ^e^. Downgraded by one level due to imprecision: confidence interval crosses the assumed threshold of clinically important difference; ^f^. Downgraded by two levels due to imprecision: small study population or very rare events; ^g^. Downgraded by one level due to publication bias: asymmetry of funnel plot with dominant positive results.

**Table 3:** Summary of findings: defecatory, urinary, and sexual dysfunction between high ligation and low ligation in colorectal cancer surgery (short-term).

Patient or population: Patients who underwent colorectal cancer surgery Setting: Randomized controlled trials Intervention: High ligation Comparison: Low ligation						
Outcomes	Number of Participants (Studies)	Certainty of the Evidence (GRADE)	Relative Effect (95% CI)	Anticipated Absolute Effects		What Happened?
				Risk with Low Ligation	Risk Difference with High Ligation	
Defecatory dysfunction (incontinence) assessed with the JWIS Scale from 0 (best) to 20 (worst) Follow-up: range of 3 to 6 months MCID: 1 points ^a^	307 (4 RCTs)	⨁⨁◯◯ LOW ^b,c^	-	JWIS ranged from 0.17 to 4.3	MD: **0.42 higher** (0.2 higher to 0.63 higher)	There may be little to no difference in defecatory dysfunction (incontinence) between HL and LL
Defecatory dysfunction (constipation) assessed with the AWCS Scale from 0 (best) to 30 (worst) Follow-up: 6 months MCID: 1.5 points ^a^	102 (2 RCTs)	⨁⨁◯◯ LOW ^b,^^d^	-	AWCS ranged from 6.0 to 6.2	MD: **1.63 higher** (0.85 higher to 2.42 higher)	HL may reduce defecatory function (constipation)
Defecatory dysfunction (overall quality of life) assessed with the GIQLI Scale from 0 (worst) to 144 (best) Follow-up: 1 month MCID: 6.5 points ^e^	196 (1 RCT)	⨁⨁◯◯ LOW ^b,c^	-	Mean GIQLI was 133.15	MD: **1.13 lower** (3.32 lower to 1.06 higher)	There may be little to no difference in defecatory dysfunction (overall quality of life) between HL and LL
Urinary dysfunction (incontinence) assessed with the ICIQ-UI Scale from 0 (best) to 21 (worst) Follow-up: range of 1 to 6 months MCID: 4 points ^f^	242 (2 RCTs)	⨁⨁◯◯ LOW ^b,c^	-	ICIQ ranged from 0.5 to 4.76	MD: **1.44 higher** (0.7 higher to 2.17 higher)	There may be little to no difference in urinary dysfunction (incontinence) between HL and LL
Urinary dysfunction (urinary symptom) assessed with the IPSS Scale from 0 (best) to 35 (worst) Follow-up: 1 month MCID: 3 points ^g^	196 (1 RCT)	⨁⨁◯◯ LOW ^b,d^	-	Mean IPSS was 20.12	MD: **1.69 higher** (0.27 lower to 3.65 higher)	There may be little to no difference in urinary dysfunction (urinary symptom) between HL and LL
Sexual dysfunction (male) assessed with the IIEF-5 Scale from 1 (worst) to 25 (best) Follow-up: range of 1 to 6 months MCID: 5 points ^h^	158 (2 RCTs)	⨁⨁◯◯ LOW ^b,d^	-	IIEF ranged from 13 to 16.41	MD: **3.73 lower** (5.46 lower to 2.01 lower)	There may be little to no difference in male sexual dysfunction between HL and LL
Sexual dysfunction (female) assessed with the FSFI Scale from 2 (worst) to 36 (best) Follow-up: 6 months MCID: 4.6 points ^i^	46 (1 RCT)	⨁⨁◯◯ LOW ^b,d^		Mean FSFI was 17	MD: **5 lower** (7.03 lower to 2.97 lower)	HL may reduce female sexual function compared with LL
**The risk in the intervention group** (and its 95% CI) is based on the assumed risk in the comparison group and the **relative effect** of the intervention (and its 95% CI). **CI:** confidence interval; **FIQL:** Fecal Incontinence Quality of Life Scale; **MCID:** minimal clinically important difference; **RCT:** randomized controlled trial; **MD:** mean difference; **HL:** high ligation; **LL:** low ligation; **JWIS:** Jorge-Wexner Incontinence Score; **AWCS:** Agachan-Wexner Constipation Score; **GIQLI:** Gastrointestinal Quality of Life Index; **ICIQ-UI:** International Consultation on Incontinence Questionnaire—Urinary Incontinence; **IPSS:** International Prostate Symptom Score; **IIEF-5:** International Index of Erectile Function-5; **FSFI:** Female Sexual Function Index						
**GRADE Working Group grades of evidence** **High certainty:** We are very confident that the true effect lies close to that of the estimate of the effect **Moderate certainty:** We are moderately confident in the effect estimate: The true effect is likely to be close to the estimate of the effect, but there is a possibility that it is substantially different **Low certainty:** Our confidence in the effect estimate is limited: The true effect may be substantially different from the estimate of the effect **Very low certainty:** We have very little confidence in the effect estimate: The true effect is likely to be substantially different from the estimate of the effect						

^a^. MCID: 25% improvement (greater than 1 point) from the baseline (HL: number; LL: number). ^b^. Downgraded by one level due to study limitations: high or unclear risk of performance and detection bias. ^c^. Downgraded by one level due to imprecision: optimal information size was not met. ^d^. Downgraded by one level due to imprecision: confidence interval crosses the assumed threshold for a clinically important difference. ^e^. MCID: from Shi et al. [37]. ^f^. MCID: from Lim et al. [38]. ^g^. MCID: from Barry et al. [39]. ^h^. MCID: from Spaliviero et al. [40]. ^i^. MCID: from Krychman et al. [41].

**Table 4:** Summary of findings: Defecatory, urinary, and sexual dysfunction between high ligation and low ligation in colorectal cancer surgery.

Patient or population: Patients who underwent colorectal cancer surgery Setting: Randomized controlled trials Intervention: High ligation Comparison: Low ligation						
Outcomes	Number of Participants (Studies)	Certainty of the Evidence (GRADE)	Relative Effect (95% CI)	Anticipated Absolute Effects		What Happened?
				Risk with Low Ligation	Risk Difference with High Ligation	
Defecatory dysfunction (incontinence) assessed with the JWIS Scale from 0 (best) to 20 (worst) Follow-up: 12 months MCID: 1 points ^a^	295 (4 RCTs)	⨁⨁◯◯ LOW ^b,c^	-	JWIS ranged from 0.10 to 3.8	MD: 0.11 higher (0.25 lower to 0.47 higher)	There may be little to no difference in defecatory dysfunction (incontinence) between HL and LL
Defecatory dysfunction (constipation) assessed with the AWCS Scale from 0 (best) to 30 (worst) Follow-up: 12 months MCID: 1.5 points ^a^	102 (2 RCTs)	⨁⨁◯◯ LOW ^b,d^	-	Mean AWCS was 6	MD: 1.61 higher (0.83 higher to 2.39 higher)	HL may reduce defecatory function (constipation) compared with LL
Defecatory dysfunction (overall quality of life) assessed with the GIQLI Scale from 0 (worst) to 144 (best) Follow-up: 9 months MCID: 6.5 points ^e^	196 (1 RCT)	⨁⨁◯◯ LOW ^b,c^	-	Mean GIQLI was 137.15	MD: 4.3 lower (6.34 lower to 2.26 lower)	There may be little to no difference in defecatory dysfunction (overall quality of life) between HL and LL
Urinary dysfunction (incontinence) assessed with the ICIQ-UI Scale from 0 (best) to 21 (worst) Follow-up: range of 9 to 12 months MCID: 4 points ^f^	242 (2 RCTs)	⨁⨁◯◯ LOW ^b,c^	-	ICIQ-UI ranged from 0.6 to 4.34	MD: 1.90 higher (0.82 higher to 2.99 higher)	There may be little to no difference in urinary dysfunction (incontinence) between HL and LL
Urinary dysfunction (urinary symptoms) assessed with the IPSS Scale from 0 (best) to 35 (worst) Follow-up: 9 months MCID: 3 points ^g^	196 (1 RCT)	⨁⨁◯◯ LOW ^b,d^	-	Mean IPSS was 18.82	MD: 4.72 higher (2.43 higher to 7.01 higher)	HL may aggravate urinary symptoms compared with LL
Sexual dysfunction (male) assessed with the IIEF-5 Scale from 1 (worst) to 25 (best) Follow-up: 9 to 12 months MCID: 5 points ^h^	158 (2 RCTs)	⨁⨁◯◯ LOW ^b,d^	-	IIEF ranged from 13 to 17.76	MD: 5.11 lower (6.85 lower to 3.37 lower)	HL may reduce male erectile function compared with LL
Sexual dysfunction (female) assessed with the FSFI Scale from 2 (worst) to 36 (best) Follow-up: 12 months MCID: 4.6 points ^i^	46 (1 RCT)	⨁⨁◯◯ LOW ^b,d^	-	Mean FSFI was 18	MD: 5 lower (6.74 lower to 3.26 lower)	HL may reduce female sexual function compared with LL
**The risk in the intervention group** (and its 95% CI) is based on the assumed risk in the comparison group and the **relative effect** of the intervention (and its 95% CI). **CI:** confidence interval; **FIQL:** Fecal Incontinence Quality of Life Scale; **MCID:** minimal clinically important difference; **RCT:** randomized controlled trial; **MD:** mean difference; **HL:** high ligation; **LL:** low ligation; **JWIS:** Jorge-Wexner Incontinence Score; **AWCS:** Agachan-Wexner Constipation Score; **GIQLI:** Gastrointestinal Quality of Life Index; **ICIQ-UI:** International Consultation on Incontinence Questionnaire—Urinary Incontinence; **IPSS:** International Prostate Symptom Score; **IIEF-5:** International Index of Erectile Function-5; **FSFI:** Female Sexual Function Index						
**GRADE Working Group grades of evidence** **High certainty:** We are very confident that the true effect lies close to that of the estimate of the effect **Moderate certainty:** We are moderately confident in the effect estimate: The true effect is likely to be close to the estimate of the effect, but there is a possibility that it is substantially different **Low certainty:** Our confidence in the effect estimate is limited: The true effect may be substantially different from the estimate of the effect **Very low certainty:** We have very little confidence in the effect estimate: The true effect is likely to be substantially different from the estimate of the effect						

^a^. MCID: 25% improvement (greater than 1 point) from the baseline (HL: number; LL: number). ^b^. Downgraded by one level due to study limitations: high or unclear risk of performance and detection bias. ^c^. Downgraded by one level due to imprecision: optimal information size was not met. ^d^. Downgraded by one level due to imprecision: confidence interval crosses the assumed threshold for a clinically important difference. ^e^. MCID: from Shi et al. [37]. ^f^. MCID: from Lim et al. [38]. ^g^. MCID: from Barry et al. [39]. ^h^. MCID: from Spaliviero et al. [40]. ^i^. MCID: from Krychman et al. [41].

## Data Availability

Not applicable.

## Supplementary Materials

The following supporting information can be downloaded at: https://www.mdpi.com/article/10.3390/medicina58091143/s1; Table S1: PRISMA 2020 for abstracts checklist.; Table S2: PRISMA 2020 checklist [57]; Table S3: Search strategy [57]; Table S4: Characteristics of the included studies (ordered by study ID); Table S5: Details of contact with the correspondents of the included trials; Table S6: Characteristics of the excluded studies.
