# Supplementary material for: High Versus Low Ligation of the Inferior Mesenteric Artery in Colorectal Cancer Surgery: A Systematic Review and Meta-Analysis
Source: Medicina (Kaunas). 2022 Aug 23;58(9):1143. doi: 10.3390/medicina58091143 (PMC9506533; doi:10.3390/medicina58091143)
Supplement: Supplementary file 1 [file medicina-58-01143-s001.zip › supplementary_file_3_search_strategy.pdf]

Table S3. Search strategy

Medline via Ovid
1. exp Colorectal Neoplasms/ or exp Colonic Neoplasms/ or exp Rectal Neoplasms/ or exp Sigmoid Neoplasms/<br>2. ((cancer* or carcinoma* or neoplasm* or adenoma* or adenocarcinom* or tumour* or tumor* or malignan*) adj3 (colorectal* or colon* or rect* or sigmoid colon*)).tw.<br>3. 1 or 2<br>4. exp Mesenteric Artery, Inferior/<br>5. (Inferior mesenteric arter* or left colic artery).tw.<br>6. ((High or Low) adj3 (Ligation or Tie)).tw.<br>7. 4 or 5 or 6<br>8. 3 and 7<br>9. Exp animals/ not humans.sh<br>10. 8 not 9
Embase (via Elsevier)
1. 'rectum tumor'/exp OR 'colon tumor'/exp OR 'colorectal tumor'/exp<br>2. ((cancer* OR carcinoma* OR neoplasm* OR adenoma* OR adenocarcinom* OR tumour* OR tumor* OR malignan*) NEAR/3 (colorectal* OR colon* OR rect* OR 'sigmoid colon*')):ti,ab<br>3. #1 OR #2<br>4. 'inferior mesenteric artery'/exp<br>5. 'inferior mesenteric arter*':ti,ab OR 'left colic artery':ti,ab<br>6. ((high OR low) NEAR/3 (ligation OR tie)):ti,ab<br>7. #4 OR #5 OR #6

8. #3 AND #7<br>9. 'animals'/exp NOT ('humans'/exp AND 'animals'/exp)<br>10. #8 NOT #9
Cochrane via Wiley
1. MeSH descriptor: [Colorectal Neoplasms] explode all trees<br>2. MeSH descriptor: [Colonic Neoplasms] explode all trees<br>3. MeSH descriptor: [Rectal Neoplasms] explode all trees<br>4. MeSH descriptor: [Sigmoid Neoplasms] explode all trees<br>5. (((cancer* or carcinoma* or neoplasm* or adenoma* or adenocarcinom* or tumour* or tumor* or malignan*) near/3 (colorectal* or colon* or rect* or "sigmoid colon*"))):ti,ab,kw (Word variations have been searched)<br>6. #1 or #2 or #3 or #4 or #5<br>7. MeSH descriptor: [Mesenteric Artery, Inferior] explode all trees<br>8. (("Inferior mesenteric arter*" or "left colic artery")):ti,ab,kw (Word variations have been searched)<br>9. (((High or Low) near/3 (Ligation or Tie))):ti,ab,kw (Word variations have been searched)<br>10. #7 or #8 or #9<br>11. #6 and #10
Scopus
TITLE-ABS-KEY((cancer* OR carcinoma* OR neoplasm* OR adenoma* OR adenocarcinom* OR tumour* OR tumor* OR malignan*) W/3 (colorectal* OR colon* OR rect* OR "sigmoid colon*")) AND TITLE-ABS-KEY("Inferior mesenteric arter*" OR "left colic artery" OR ((high OR low) W/3 (ligation OR tie)))

AND NOT INDEX (medline)
Web of Science
1. TS=((cancer* OR carcinoma* OR neoplasm* OR adenoma* OR adenocarcinom* OR tumour* OR tumor* OR malignan*) NEAR/3 (colorectal* OR colon* OR rect* OR "sigmoid colon*")) AND TS=("Inferior mesenteric arter*" OR "left colic artery" OR ((high OR low) NEAR/3 (ligation OR tie)))
LILACS
1. (MH:"Colorectal Neoplasms" OR tw:((colorectal$ OR colon$ OR rect$ OR "sigmoid colon") and (cancer$ OR carcinoma$ OR neoplasm$ OR adenoma$ OR adenocarcinom$ OR tumour$ OR tumor$ OR malignan$))) AND (MH:"Mesenteric Artery, Inferior" OR tw:("Inferior mesenteric arter*" OR "left colic artery" OR "high ligation" OR "low ligation" OR "high tie" OR "low tie")) AND NOT (MH:animals OR MH:rabbits OR MH:rats OR MH:primates OR MH:dogs OR MH:cats OR MH:swine OR PT:"in vitro")
ClinicalTrials.gov
1. Colorectal cancer<br>2. Inferior Mesenteric Arter*<br>3. 1 AND 2
World Health Organization International Clinical Trials Registry Platform search portal
1. Colorectal AND Inferior Mesenteric Arter*
Grey Literature (Open Grey)
1. Colorectal AND Inferior Mesenteric Arter*
