# Supplementary material for: High Versus Low Ligation of the Inferior Mesenteric Artery in Colorectal Cancer Surgery: A Systematic Review and Meta-Analysis
Source: Medicina (Kaunas). 2022 Aug 23;58(9):1143. doi: 10.3390/medicina58091143 (PMC9506533; doi:10.3390/medicina58091143)
Supplement: Supplementary file 1 [file medicina-58-01143-s001.zip › supplementary_file_4_cha_inc_studies.pdf]

Table S4. Characteristics of included studies (ordered by study ID)

A. Study ID for included studies

Study ID	Identified articles
Feng 2021	* Feng W, Zong Y, Zhao J, Li W, Miao Y, Xu Z, et al. High versus low ligation of the inferior mesenteric artery during laparoscopic rectal cancer surgery: A prospective study of surgical and oncological outcomes. Journal of Surgical Oncology 2021.<br>• Sun J, Feng W, Yu C, Xue P, Lu A, Zheng M. Lca-preserving technique reduces the anastomotic leakage rate in laparoscopic mid/low rectal cancer surgery: Midterm results from a single-center randomized controlled trial. Surgical Endoscopy and Other Interventional Techniques 2018;32(1):S208.
Flori 2020	* Fiori E, Crocetti D, Lamazza A, De Felice F, Scotti G B, Sterpetti A V, et al. Defecatory dysfunction after colon cancer resection: The role of inferior mesenteric artery tie. Anticancer Research 2020;40(5):2969-74.
Flori 2020a	* Fiori E, Crocetti D, Lamazza A, De Felice F, Sterpetti A V, Irace L, et al. Is low inferior mesenteric artery ligation worthwhile to prevent urinary and sexual dysfunction after total mesorectal excision for rectal cancer? Anticancer Research 2020;40(8):4223-8.
Fujii 2018	* Fujii S, Ishibe A, Ota M, Watanabe K, Watanabe J, Kunisaki C, et al. Randomized clinical trial of high versus low inferior mesenteric artery ligation during anterior resection for rectal cancer. BJS open 2018;2(4):195-202.<br>• Nct. Randomized Controlled Trial to Evaluate High Tie Versus Low Tie of the Inferior Mesenteric Artery in Anterior Resection. https://clinicaltrials.gov/show/NCT01861678 2013.<br>• Shoichi Fujii MD PhD, Center Yokohama City University Medical. Randomized Controlled Trial to Evaluate High Tie Versus Low Tie of the Inferior Mesenteric Artery in Anterior Resection. 2006.
Guo 2017	* Guo Y, Wang D, He L, Zhang Y, Zhao S, Zhang L, et al. Marginal artery stump pressure in left colic artery-preserving rectal cancer surgery: a clinical trial. ANZ journal of surgery 2017;87(7):576-81.<br>• Suo Jian, University The First Hospital of Jilin. Study of the Preservation of the Left Colic Artery on Rectum Cancer Surgery. 2013.
Kruszewski 2021	* Wiesław Janusz Kruszewski, Mariusz Szajewski, MaciejCiesielski, Tomasz Buczek, Krzysztof Kawecki, Jakub Walczak. Level of inferior mesenteric artery ligation does not affect rectal cancer treatment outcomes inspite of better cancer specific survival after low ligation – randomized trial results. Colorectal disease. July 2021.<br>• Kruszewski W J, Ciesielski M, Szajewski M, Jasinski W, Szefel J, Kawecki K, et al. Level of arterial ligation and early results of rectal cancer surgery-Preliminary report of randomized trial. European Journal of Surgical Oncology 2012;38(9):759.<br>• Isrctn. Level of arterial ligation in rectal cancer surgery. http://www.who.int/trialsearch/Trial2.aspx?TrialID=ISRCTN12189372 2012.
Mari 2019	* Mari G M, Crippa J, Cocozza E, Berselli M, Livraghi L, Carzaniga P, et al. Low Ligation of Inferior Mesenteric Artery in Laparoscopic Anterior Resection for Rectal Cancer Reduces Genitourinary Dysfunction: Results From a Randomized Controlled Trial (HIGHLOW Trial). Annals of surgery 2019;269(6):1018-24.<br>• Costanzi A, Mari G, Crippa J, Maggioni D. Low ligation of IMA in laparoscopic anterior resection for rectal cancer reduces genitourinary dysfunction. Surgical Endoscopy 2019;33(2):S503.<br>• Mari G, Maggioni D, Costanzi A, Mir, a A, Rigamonti L, et al. "High or low Inferior Mesenteric Artery ligation in Laparoscopic low Anterior Resection: Study protocol for a randomized controlled trial" (HIGHLOW trial). Trials 2015;16(1).

Matsuda 2015, 2017	* Matsuda K, Yokoyama S, Hotta T, Takifuji K, Watanabe T, Tamura K, et al. Oncological outcomes following rectal cancer surgery with high or low ligation of the inferior mesenteric artery. Gastrointestinal Tumors 2017;4(1):45-52.<br>• Matsuda K, Hotta T, Takifuji K, Yokoyama S, Oku Y, Watanabe T, et al. Randomized clinical trial of defaecatory function after anterior resection for rectal cancer with high versus low ligation of the inferior mesenteric artery. British Journal of Surgery 2015;102(5):501-8.<br>• Matsuda K, Hotta T, Takifuji K, Yokoyama S, Oku Y, Yamaue H. Evaluation of defecatory function following anterior resection for rectal cancer: Prospective, randomized-controlled trial of high tie or low tie. Diseases of the Colon and Rectum 2014;57(5):e321.<br>• Nct. Comparison of Low and High Ligation in the Rectal Cancer. https://clinicaltrials.gov/show/NCT00701012 2008.<br>• Nct. Comparison of Low and High Ligation in the Rectal Cancer. https://clinicaltrials.gov/show/NCT00701012 2008.
Niu 2016	* Niu J, Ning W, Wang W, Pei D, Meng F, Liu Z, et al. Clinical effect of preservation of the left colonic artery in laparoscopic anterior resection for rectal cancer. National Medical Journal of China 2016;96(44):3582-5.
Wang 2015	* Wang Q, Zhang C, Zhang H, Wang Y, Yuan Z, Di C. Effect of ligation level of inferior mesenteric artery on postoperative defecation function in patients with rectal cancer. Zhonghua wei chang wai ke za zhi = Chinese journal of gastrointestinal surgery 2015;18(11):1132-5.
Wu 2017	* Ying-Jun Wu, Min Li. Clinical research of laparoscopic low anterior resection with preservation of the left colonic artery. Chin J Gastrointest Surg November 2017;20(11):1313-1315.
Zhou 2018	* Zhou J, Zhang S, Huang J, Huang P, Peng S, Lin J, et al. Accurate low ligation of inferior mesenteric artery and root lymph node dissection according to different vascular typing in laparoscopic radical resection of rectal cancer. Zhonghua wei chang wai ke za zhi = Chinese journal of gastrointestinal surgery 2018;21(1):46-52.

* This is the primary reference for the study

B. Feng 2021

Study characteristics	
Methods	**Study design:** prospective, randomized, parallel study<br>**Setting/country:** likely single center/China<br>**Date when study was conducted:** October 2016 to September 2018
Participants	**Inclusion criteria**<br>• Histologically proven adenocarcinoma<br>• Rectal lesion (distal margin 5–15 cm from the anus)<br>• Clinical stages I–III disease (based on computed tomography or magnetic resonance imaging)<br>• A Karnofsky score of ≥80 (unable to perform strenuous physical activity but ambulatory and able to perform light or sedentary work)<br>• 18–75 years old.<br><br>**Exclusion criteria**<br>• History of other malignancies<br>• Received preoperative chemotherapy and/or radiotherapy, or<br>• Underwent emergency surgery because of bowel perforation, bleeding, or obstruction<br><br>**Total number of participants randomly assigned:** 95<br><br>**High ligation**<br>• Number of all participant randomly assigned: 47<br>**Low ligation**<br>• Number of all participant randomly assigned: 48
Interventions	**Intervention:** high ligation<br>**Comparator:** low ligation<br>**Follow-up:** 24 months
Outcomes	**Outcomes**<br>• Operation time<br>• Intraoperative blood loss<br>• Intraoperative adverse events (extended bowel excision)<br>• Post-operative anastomosis-related complications (anastomosis site leakage, stenosis, bleeding)<br>• Distances to the proximal and distal margins<br>• Number of harvested lymph nodes (number of positive lymph nodes)<br>• Number of IMA vascular root lymph nodes<br>• Cancer-related mortality<br>• Overall survival<br>• Recurrence, metastasis
Funding sources	Not reported
Declarations of interest	None
Notes	**Protocol:** ChiCTR-INR-17010336<br>**Language of publication:** English

Risk of bias		
Bias	Authors' judgement	Support for judgement
Random sequence generation (selection bias)	Low risk	Quote: "Patient information was sent to a third-party organization for randomization on the day of surgery"
Allocation concealment (selection bias)	Unclear risk	Judgement: not described
Blinding of participants and personnel (performance bias)<br>: All outcomes	High risk	Quote: "The surgeons were not blinded"
Blinding of outcome assessment	High risk	Quote: "The surgeons were not blinded"

(detection bias)<br>: Subjective outcomes		
Blinding of outcome assessment (detection bias)<br>: Objective outcomes	Low risk	Judgement: objective outcomes were unlikely affected by lack of blinding.
Incomplete outcome data (attrition bias)<br>: Overall mortality	Low risk	Judgement: all participants were included in the analysis
Incomplete outcome data (attrition bias)<br>: Postoperative complication	Low risk	Judgement: all participants were included in the analysis
Incomplete outcome data (attrition bias)<br>: Disease recurrence	Low risk	Judgement: all participants were included in the analysis
Incomplete outcome data (attrition bias)<br>: Cancer specific mortality	Unclear risk	Judgement: No information (not measured)
Incomplete outcome data (attrition bias)<br>: Postoperative mortality	Unclear risk	Judgement: No information (not measured)
Incomplete outcome data (attrition bias)<br>: Anastomotic leakage	Low risk	Judgement: all participants were included in the analysis
Incomplete outcome data (attrition bias)<br>: Urinary dysfunction assessed by the ICIQ-UI and/or the IPSS	Unclear risk	Judgement: no information (not measured)
Incomplete outcome data (attrition bias)<br>: Sexual dysfunction assessed by the IIEF and/or the FSFI	Unclear risk	Judgement: no information (not measured)
Incomplete outcome data (attrition bias)<br>: Defecatory dysfunction assessed by the FIQL, the JWIS, the AWCS, and the GIQLI	Unclear risk	Judgement: no information (not measured)
Selective reporting (reporting bias)	Low risk	Judgement: not detected
Other source of bias	Low risk	Judgement: not detected

IMA, inferior mesenteric artery, ICIQ-UI, international consultation on incontinence questionnaire urinary incontinence, IPSS, international prostate symptom score, IIEF, international index of erectile function, FSFI, female sexual function index, FIQL, fecal incontinence quality of life, JWIS, Jorge-Wexner incontinence score, AWCS, Agachan-Wexner constipation score, GIQLI, gastrointestinal quality of life index

C. Flori 2020

Study characteristics	
Methods	**Study design:** prospective randomized open label parallel trial<br>**Setting/country:** single center/Italy<br>**Date when study was conducted:** February 2013 to December 2018
Participants	**Inclusion criteria**<br>• Stage II<br>• Stage III<br>• M0<br>• Sigmoidal cancer<br>• Laparoscopic surgery<br>**Exclusion criteria**<br>• Stage I<br>• Stage IV<br>• Emergency surgery<br>• Previous stenting for colonic obstruction.<br>• Metastatic disease<br>• Laparotomic approach<br>• Conversion to laparotomy<br>• History of fecal incontinence<br>**Total number of participants randomly assigned:** 56<br><br>**High ligation**<br>• Number of all participant randomly assigned: 32<br>**Low ligation**<br>• Number of all participant randomly assigned: 24
Interventions	**Intervention:** high ligation<br>**Comparator:** low ligation<br>**Follow-up:** 60 months
Outcomes	Early results<br>• Postoperative mortality<br>• Major complication<br>• Minor complication (urinary bladder retention, postoperative vomitting, wound infection)<br>• Oral feeding resume<br>• Overall length of stay<br>Long-term results<br>• Overall survival rate<br>• Disease-free survival<br>Defecatory outcome<br>• Fecal incontinence quality of life score<br>• Jorge-Wexner incontinence score<br>• Agachan-Wexner constipation score
Funding sources	Not reported
Declarations of interest	None
Notes	**Protocol:** NCT03451643<br>**Language of publication:** English

Risk of bias		
Bias	Authors' judgement	Support for judgement
Random sequence generation (selection bias)	Unclear risk	Judgement: not described
Allocation concealment (selection bias)	Unclear risk	Judgement: not described
Blinding of participants and personnel (performance bias)	High risk	Quote: "open label"

: All outcomes		
Blinding of outcome assessment (detection bias)<br>: Subjective outcomes	High risk	Quote: "open label"
Blinding of outcome assessment (detection bias)<br>: Objective outcomes	Low risk	Judgement: objective outcomes were unlikely affected by lack of blinding.
Incomplete outcome data (attrition bias)<br>: Overall survival	Low risk	Judgement: all participants were included in the analysis
Incomplete outcome data (attrition bias)<br>: Postoperative complication	Low risk	Judgement: all participants were included in the analysis
Incomplete outcome data (attrition bias)<br>: Disease recurrence	Low risk	Judgement: all participants were included in the analysis
Incomplete outcome data (attrition bias)<br>: Cancer specific mortality	Unclear risk	Judgement: no information (not measured)
Incomplete outcome data (attrition bias)<br>: Postoperative mortality	Low risk	Judgement: all participants were included in the analysis
Incomplete outcome data (attrition bias)<br>: Anastomotic leakage	Unclear risk	Judgement: no information (not measured)
Incomplete outcome data (attrition bias)<br>: Urinary dysfunction assessed by the ICIQ-UI and/or the IPSS	Unclear risk	Judgement: no information (not measured)
Incomplete outcome data (attrition bias)<br>: Sexual dysfunction assessed by the IIEF and/or the FSFI	Unclear risk	Judgement: no information (not measured)
Incomplete outcome data (attrition bias)<br>: Defecatory dysfunction assessed by the FIQL, the JWIS, the AWCS, and the GIQLI	Low risk	Judgement: all participants were included in the analysis
Selective reporting (reporting bias)	High risk	Judgement: variables associated operative outcome in material and methods but not in results
Other source of bias	High risk	Judgement: protocol was published, but the content of the protocol was not identical to published full-text article

IMA, inferior mesenteric artery, ICIQ-UI, international consultation on incontinence questionnaire urinary incontinence, IPSS, international prostate symptom score, IIEF, international index of erectile function, FSFI, female sexual function index, FIQL, fecal incontinence quality of life, JWIS, Jorge-Wexner incontinence score, AWCS, Agachan-Wexner constipation score, GIQLI, gastrointestinal quality of life index

D. Flori 2020a

Study characteristics	
Methods	**Study design:** prospective randomized open label parallel trial<br>**Setting/country:** single center/Italy<br>**Date when study was conducted:** February 2013 to March 2019
Participants	**Inclusion criteria**<br>• Curative laparoscopic resection for pT2N0M0<br>• Rectal adenocarcinoma<br>• Laparoscopic TME<br>**Exclusion criteria**<br>• T1, T3, T4 rectal cancer or N+ rectal cancer<br>• Neoadjuvant /adjevant radio-chemotherapy,<br>• Rectal cancer lower than 5cm,<br>• Emergent surgery<br>• Previous stenting for rectal obstruction<br>• Metastatic disease<br>• Laparotomic approach or conversion to laparotomy<br>• History of sexual or urinary dysfunction<br><br>**Total number of participants randomly assigned:** 46<br><br>**High ligation**<br>• Number of all participant randomly assigned: 22<br>**Low ligation**<br>• Number of all participant randomly assigned: 24
Interventions	**Intervention:** high ligation<br>**Comparator:** low ligation<br>**Follow-up:** 60 months
Outcomes	Early results<br>• Postoperative mortality<br>• Major complication<br>• Minor complication (wound infection, urinary retention, phlebitis)<br>• Oral feeding resume<br>• Overall length of stay<br>Long-term results<br>• Overall survival rate<br>• Disease-free survival<br>Defecatory, urinary and sexual function outcome assessed by<br>• Fecal incontinence quality of life score<br>• Jorge-Wexner incontinence score<br>• Agachan-Wexner constipation score<br>• International consultation on incontinence questionnaire urinary incontinence short form<br>• Female sexual function index<br>• International index of erectile function
Funding sources	Not reported
Declarations of interest	None
Notes	**Protocol:** NCT03451643<br>**Language of publication:** English

Risk of bias		
Bias	Authors' judgement	Support for judgement
Random sequence generation (selection bias)	Unclear risk	Judgement: not described
Allocation concealment (selection bias)	Unclear risk	Judgement: not described
Blinding of participants and	High risk	Quote: "open label"

personnel (performance bias)<br>: All outcomes		
Blinding of outcome assessment (detection bias)<br>: Subjective outcomes	High risk	Quote: "open label"
Blinding of outcome assessment (detection bias)<br>: Objective outcomes	Low risk	Judgement: objective outcomes were unlikely affected by lack of blinding.
Incomplete outcome data (attrition bias)<br>: Overall mortality	Low risk	Judgement: all participants were included in the analysis
Incomplete outcome data (attrition bias)<br>: Postoperative complication	Low risk	Judgement: all participants were included in the analysis
Incomplete outcome data (attrition bias)<br>: Disease recurrence	Low risk	Judgement: all participants were included in the analysis
Incomplete outcome data (attrition bias)<br>: Cancer specific mortality	Unclear risk	Judgement: no information (not measured)
Incomplete outcome data (attrition bias)<br>: Postoperative mortality	Low risk	Judgement: all participants were included in the analysis
Incomplete outcome data (attrition bias)<br>: Anastomotic leakage	Unclear risk	Judgement: no information (not measured)
Incomplete outcome data (attrition bias)<br>: Urinary dysfunction assessed by the ICIQ-UI and/or the IPSS	Low risk	Judgement: all participants were included in the analysis
Incomplete outcome data (attrition bias)<br>: Sexual dysfunction assessed by the IIEF and/or the FSFI	Low risk	Judgement: all participants were included in the analysis
Incomplete outcome data (attrition bias)<br>: Defecatory dysfunction assessed by the FIQL, the JWIS, the AWCS, and the GIQLI	Low risk	Judgement: all participants were included in the analysis
Selective reporting (reporting bias)	High risk	Judgement: variables associated operative outcome in material and methods but not in results
Other source of bias	High risk	Judgement: protocol was published, but the content of the protocol was not identical to published full-text article

TME, total mesorectal excision, ICIQ-UI, international consultation on incontinence questionnaire urinary incontinence, IPSS, international prostate symptom score, IIEF, international index of erectile function, FSFI, female sexual function index, FIQL, fecal incontinence quality of life, JWIS, Jorge-Wexner incontinence score, AWCS, Agachan-Wexner constipation score, GIQLI, gastrointestinal quality of life index

E. Fujii 2018

Study characteristics	
Methods	**Study design:** randomized controlled trial, open label method<br>**Setting/country:** single center/Japan<br>**Date when study was conducted:** June 2006 to September 2012
Participants	**Inclusion criteria**<br>• Age 20 years or above<br>• Histologically proven adenocarcinoma of the rectum.<br>**Exclusion criteria**<br>• A primary tumour that directly invaded another organ clinically (T4b)<br>• Synchronous distant or peritoneal metastasis<br>• Operation scheduled as an emergency<br>• Previous history of colorectal surgery except for appendicectomy<br>• Active or recent treatment for malignancy in another organ<br>• Multiple colorectal cancers that needed construction of two or more anastomoses<br>• Pregnant and lactating women were excluded<br>• Patients scheduled for resection without colorectal anastomosis<br><br>**Total number of participants randomly assigned:** 324<br><br>**High ligation**<br>• Number of all participant randomly assigned: 164<br>**Low ligation**<br>• Number of all participant randomly assigned: 160
Interventions	**Intervention:** high ligation<br>**Comparator:** low ligation<br>**Follow-up:** 60 months
Outcomes	**Primary outcome**<br>• Anastomotic leakage<br>**Secondary outcome**<br>• Duration of surgery<br>• Blood loss<br>• 5-year overall survival rate.
Funding sources	Not reported
Declarations of interest	None
Notes	**Protocol:** NCT01861678<br>**Language of publication:** English

Risk of bias		
Bias	Authors' judgement	Support for judgement
Random sequence generation (selection bias)	Low risk	Quote: "The randomization was performed by the department of biostatistics and epidemiology data center of Yokohama city university immediately before the operation"
Allocation concealment (selection bias)	Unclear risk	Judgement: not described
Blinding of participants and personnel (performance bias)<br>: All outcomes	High risk	Quote: "open label"
Blinding of outcome assessment (detection bias)<br>: Subjective outcomes	High risk	Quote: "open label"
Blinding of outcome assessment (detection bias)<br>: Objective outcomes	Low risk	Judgement: objective outcomes were unlikely affected by lack of blinding.
Incomplete outcome data (attrition bias)	Low risk	Judgement: all participants were included in the analysis

: Overall mortality		
Incomplete outcome data (attrition bias)<br>: Postoperative complication	Low risk	Judgement: all participants were included in the analysis
Incomplete outcome data (attrition bias)<br>: Disease recurrence	Low risk	Judgement: all participants were included in the analysis
Incomplete outcome data (attrition bias)<br>: Cancer specific mortality	Unclear risk	Judgement: no information (not measured)
Incomplete outcome data (attrition bias)<br>: Postoperative mortality	Low risk	Judgement: all participants were included in the analysis
Incomplete outcome data (attrition bias)<br>: Anastomotic leakage	Low risk	Judgement: all participants were included in the analysis
Incomplete outcome data (attrition bias)<br>: Urinary dysfunction assessed by the ICIQ-UI and/or the IPSS	Unclear risk	Judgement: no information (not measured)
Incomplete outcome data (attrition bias)<br>: Sexual dysfunction assessed by the IIEF and/or the FSFI	Unclear risk	Judgement: no information (not measured)
Incomplete outcome data (attrition bias)<br>: Defecatory dysfunction assessed by the FIQL, the JWIS, the AWCS, and the GIQLI	Unclear risk	Judgement: no information (not measured)
Selective reporting (reporting bias)	Low risk	Judgement: not detected
Other source of bias	Low risk	Judgement: not detected

ICIQ-UI, international consultation on incontinence questionnaire urinary incontinence, IPSS, international prostate symptom score, IIEF, international index of erectile function, FSFI, female sexual function index, FIQL, fecal incontinence quality of life, JWIS, Jorge-Wexner incontinence score, AWCS, Agachan-Wexner constipation score, GIQLI, gastrointestinal quality of life index

F. Guo 2017

Study characteristics	
Methods	**Study design:** interventional randomized, single (subject) blinded parallel trial<br>**Setting/country:** single center/China<br>**Date when study was conducted:** February 2013 to December 2013
Participants	**Inclusion criteria**<br>• Only solitary radical resectable rectal cancers 3–20 cm from the anus<br>• The rectal cancer was their first malignant neoplasm<br>**Exclusion criteria**<br>• Patients with any distant metastasis detected before or during the operation<br>• Patients receiving steroid medication or preoperative radiotherapy<br><br>**Total number of participants randomly assigned:** 57<br><br>**High ligation**<br>• Number of all participant randomly assigned: 29<br>**Low ligation**<br>• Number of all participant randomly assigned: 28
Interventions	**Intervention:** high ligation<br>**Comparator:** low ligation<br>**Follow-up:** Not reported
Outcomes	**Primary outcome**<br>• The blood pressure of the arterial arcade<br>• Anastomotic leakage<br>**Secondary outcome**<br>• Distal colon length<br>• OP time<br>• Lymph node retrieval rate
Funding sources	The Health Project of the Jilin Province, China
Declarations of interest	None
Notes	**Protocol:** NCT01979029<br>**Language of publication:** English

Risk of bias		
Bias	Authors' judgement	Support for judgement
Random sequence generation (selection bias)	High risk	Quote: "Using a computer-generated random number table, with odd numbers resulting in allocation of a patient to group A and even numbers leading to inclusion of the patient in group B"
Allocation concealment (selection bias)	Unclear risk	Judgement: not described
Blinding of participants and personnel (performance bias)<br>: All outcomes	High risk	Quote: single(subject) blinded
Blinding of outcome assessment (detection bias)<br>: Subjective outcomes	High risk	Quote: single(subject) blinded
Blinding of outcome assessment (detection bias)<br>: Objective outcomes	Low risk	Judgement: objective outcomes were unlikely affected by lack of blinding.
Incomplete outcome data (attrition bias)<br>: Overall mortality	Unclear risk	Judgement: no information (not measured)
Incomplete outcome data (attrition bias)	Unclear risk	Judgement: no information (not measured)

: Postoperative complication		
Incomplete outcome data (attrition bias)<br>: Disease recurrence	Unclear risk	Judgement: no information (not measured)
Incomplete outcome data (attrition bias)<br>: Cancer specific mortality	Unclear risk	Judgement: no information (not measured)
Incomplete outcome data (attrition bias)<br>: Postoperative mortality	Unclear risk	Judgement: no information (not measured)
Incomplete outcome data (attrition bias)<br>: Anastomotic leakage	Low risk	Judgement: all participants were included in the analysis
Incomplete outcome data (attrition bias)<br>: Urinary dysfunction assessed by the ICIQ-UI and/or the IPSS	Unclear risk	Judgement: no information (not measured)
Incomplete outcome data (attrition bias)<br>: Sexual dysfunction assessed by the IIEF and/or the FSFI	Unclear risk	Judgement: no information (not measured)
Incomplete outcome data (attrition bias)<br>: Defecatory dysfunction assessed by the FIQL, the JWIS, the AWCS, and the GIQLI	Unclear risk	Judgement: no information (not measured)
Selective reporting (reporting bias)	High risk	Judgement: protocol was published, but study outcomes were not identical to the outcomes prespecified in the protocol
Other source of bias	Low risk	Judgement: not detected

ICIQ-UI, international consultation on incontinence questionnaire urinary incontinence, IPSS, international prostate symptom score, IIEF, international index of erectile function, FSFI, female sexual function index, FIQL, fecal incontinence quality of life, JWIS, Jorge-Wexner incontinence score, AWCS, Agachan-Wexner constipation score, GIQLI, gastrointestinal quality of life index

G. Kruszewski 2021

Study characteristics	
Methods	**Study design:** interventional randomized controlled trial, unblinded<br>**Setting/country:** single center/Poland<br>**Date when study was conducted:** April 22, 2010 to March 8, 2016
Participants	**Inclusion criteria**<br>• Patients underwent radical surgery due to rectal or rectosigmoid adenocarcinoma were recruited to the study.<br>**Exclusion criteria**<br>• Urgent operation<br>• Potentially unresectable disease<br>• Comorbid conditions excluding the possibility of standard therapy<br>• T4 tumour<br>• Synchronous distant metastases<br>• History or the presence of any other malignant neoplasm except skin Cancer<br><br>**Total number of participants randomly assigned:** 130<br><br>**High ligation**<br>• Number of all participant randomly assigned: 65<br>**Low ligation**<br>• Number of all participant randomly assigned: 65
Interventions	**Intervention:** high ligation<br>**Comparator:** low ligation<br>**Follow-up:** more than 60 months
Outcomes	**Outcomes**<br>• Overall survival, disease free survival, cancer specific survival<br>• Postoperative mortality<br>• Postoperative complication<br>• Surgical treatment - surgery duration, type of surgery, number of transfused blood units<br>• Anastomotic leak
Funding sources	Not reported
Declarations of interest	None
Notes	**Protocol:** ISRCTN12189372<br>**Language of publication:** English

Risk of bias		
Bias	Authors' judgement	Support for judgement
Random sequence generation (selection bias)	Low risk	Quote: "The option of ligation level was determined according to the consecutive number generated by Random function in Microsoft Excel (1- meant HL, 2 meant LL)."
Allocation concealment (selection bias)	Low risk	Quote: "Randomly determined numbers (1 or 2) for ligation options were placed in 130 consecutively numbered sealed, opaque envelopes opened in the operating room after making the decision to enroll the patient.
Blinding of participants and personnel (performance bias)<br>: All outcomes	High risk	Quote: "unblinded"
Blinding of outcome assessment (detection bias)<br>: Subjective outcomes	High risk	Quote: "unblinded"
Blinding of outcome assessment (detection bias)	Low risk	Judgement: objective outcomes were unlikely affected by lack of blinding.

: Objective outcomes		
Incomplete outcome data (attrition bias)<br>: Overall mortality	Low risk	Judgement: all participants were included in the analysis
Incomplete outcome data (attrition bias)<br>: Postoperative complication	Low risk	Judgement: all participants were included in the analysis
Incomplete outcome data (attrition bias)<br>: Disease recurrence	Low risk	Judgement: 59/65 (90.8%) and 59/65 (90.8%) participants randomized to high ligation and low ligation were included in the analysis, respectively
Incomplete outcome data (attrition bias)<br>: Cancer specific mortality	Low risk	Judgement: 59/65 (90.8%) and 59/65 (90.8%) participants randomized to high ligation and low ligation were included in the analysis, respectively
Incomplete outcome data (attrition bias)<br>: Postoperative mortality	Low risk	Judgement: all participants were included in the analysis
Incomplete outcome data (attrition bias)<br>: Anastomotic leakage	Low risk	Judgement: all participants were included in the analysis
Incomplete outcome data (attrition bias)<br>: Urinary dysfunction assessed by the ICIQ-UI and/or the IPSS	Unclear risk	Judgement: no information (not measured)
Incomplete outcome data (attrition bias)<br>: Sexual dysfunction assessed by the IIEF and/or the FSFI	Unclear risk	Judgement: no information (not measured)
Incomplete outcome data (attrition bias)<br>: Defecatory dysfunction assessed by the FIQL, the JWIS, the AWCS, and the GIQLI	Unclear risk	Judgement: no information (not measured)
Selective reporting (reporting bias)	High risk	Judgement: protocol was published, but study outcomes were not identical to the outcomes prespecified in the protocol
Other source of bias	Low risk	Judgement: not detected

ICIQ-UI, international consultation on incontinence questionnaire urinary incontinence, IPSS, international prostate symptom score, IIEF, international index of erectile function, FSFI, female sexual function index, FIQL, fecal incontinence quality of life, JWIS, Jorge-Wexner incontinence score, AWCS, Agachan-Wexner constipation score, GIQLI, gastrointestinal quality of life index

H. Mari 2019

Study characteristics	
Methods	**Study design:** Randomized controlled trial, open label parallel trial<br>**Setting/country:** Multi center/Italy<br>**Date when study was conducted:** June 2014 to December 2016
Participants	**Inclusion criteria**<br>• 18 years of age or older<br>• BMI less than 30<br>• ASA I, II, III<br>• Elective laparoscopic LAR +TME<br>• No evidence of metastatic disease<br>**Exclusion criteria**<br>• Prior surgery on the abdominal aorta<br>• Computed tomography-proven arteriosclerosis of the IMA and its branches<br><br>**Total number of participants randomly assigned:** 196<br><br>**High ligation**<br>• Number of all participant randomly assigned: 101<br>**Low ligation**<br>• Number of all participant randomly assigned: 95
Interventions	**Intervention:** high ligation<br>**Comparator:** low ligation<br>**Follow-up:** 12 months †
Outcomes	**Primary outcomes**<br>• Genitourinary dysfunction (Sexual and urinary function)<br>• Quality of life<br>**Secondary outcomes**<br>• Anastomotic leak<br>• Oncological outcomes: disease free survival, overall survival, local recurrence, distant metastasis
Funding sources	Not reported
Declarations of interest	None
Notes	**Protocol:** NCT02153801<br>**Language of publication:** English

Risk of bias		
Bias	Authors' judgement	Support for judgement
Random sequence generation (selection bias)	Low risk	Quote: "the random allocation was generated using a computerized randomization system and performed using sealed envelopes"
Allocation concealment (selection bias)	Low risk	Quote: "the random allocation was generated using a computerized randomization system and performed using sealed envelopes"
Blinding of participants and personnel (performance bias)<br>: All outcomes	High risk	Quote: "open label"
Blinding of outcome assessment (detection bias)<br>: Subjective outcomes	High risk	Quote: "open label"
Blinding of outcome assessment (detection bias)<br>: Objective outcomes	Low risk	Judgement: objective outcomes were unlikely affected by lack of blinding.
Incomplete outcome data (attrition bias)<br>: Overall mortality	Unclear risk	Judgement: no information (not measured)

Incomplete outcome data (attrition bias)<br>: Postoperative complication	Low risk	Judgement: all participants were included in the analysis
Incomplete outcome data (attrition bias)<br>: Disease recurrence	Low risk	Judgement: all participants were included in the analysis
Incomplete outcome data (attrition bias)<br>: Cancer specific mortality	Unclear risk	Judgement: no information (not measured)
Incomplete outcome data (attrition bias)<br>: Postoperative mortality	Low risk	Judgement: all participants were included in the analysis
Incomplete outcome data (attrition bias)<br>: Anastomotic leakage	Low risk	Judgement: all participants were included in the analysis
Incomplete outcome data (attrition bias)<br>: Urinary dysfunction assessed by the ICIQ-UI and/or the IPSS	Low risk	Judgement: all participants were included in the analysis
Incomplete outcome data (attrition bias)<br>: Sexual dysfunction assessed by the IIEF and/or the FSFI	High risk	Judgement: 62/101 (61.4%) and 50/95 (52.6%) participants randomized to high ligation and low ligation were included in the analysis using IIEF, respectively
Incomplete outcome data (attrition bias)<br>: Defecatory dysfunction assessed by the FIQL, the JWIS, the AWCS, and the GIQLI	Low risk	Judgement: all participants were included in the analysis
Selective reporting (reporting bias)	High risk	Judgement: no overall survival in Results but in material and methods
Other source of bias	Low risk	Judgement: not detected

BMI, body mass index, ASA, American Society of Anesthesiologists, LAR, low anterior resection, TME, total mesorectal excision, ICIQ-UI, international consultation on incontinence questionnaire urinary incontinence, IPSS, international prostate symptom score, IIEF, international index of erectile function, FSFI, female sexual function index, FIQL, fecal incontinence quality of life, JWIS, Jorge-Wexner incontinence score, AWCS, Agachan-Wexner constipation score, GIQLI, gastrointestinal quality of life index

† From study protocol

I. Matsuda 2015, 2017

Study characteristics	
Methods	**Study design:** Randomized controlled trial, double blinded (investigator, outcome assessor) parallel<br>**Setting/country:** single center/Japan<br>**Date when study was conducted:** February 2008 to December 2011
Participants	**Inclusion criteria**<br>• Patients were included if they were scheduled for anterior resection with reconstruction using the double-stapling technique for rectal cancer.<br>**Exclusion criteria**<br>• Presence of tumour located more than 15 cm above the anal verge<br>• Unresectable metastatic disease;<br>• Lymph node metastasis around the root of the left colic artery<br>• Inability to complete or comprehend the questionnaire.<br>• Patients with clinical Tis and T1 tumours,<br><br>**Total number of participants randomly assigned:** 100<br><br>**High ligation**<br>• Number of all participant randomly assigned: 51<br>**Low ligation**<br>• Number of all participant randomly assigned: 49
Interventions	**Intervention:** high ligation<br>**Comparator:** low ligation<br>**Follow-up:** 36 months †
Outcomes	**Matsuda 2015**<br>**Primary outcomes**<br>• Defecatory dysfunction (JWIS, FIQL, stool frequency, ability to discriminate gas from stool, need for antidiarrhoeal or laxative drugs, nocturnal leakage, pad use, sensation of incomplete evacuation, evacuation time, nocturnal bowel movements and urgency)<br>**Secondary outcomes**<br>• Postoperative morbidity<br>• Harvested lymph node status<br>• Oncological outcomes<br><br>**Matsuda 2017**<br>**Primary outcomes**<br>• Lymph node status<br>• Oncologic outcomes
Funding sources	Not reported
Declarations of interest	None
Notes	**Protocol:** NCT00701012<br>**Language of publication:** English

Risk of bias		
Bias	Authors' judgement	Support for judgement
Random sequence generation (selection bias)	Low risk	Quote: "A research physician carried out the randomization using a computer-generated random pattern (block size 4) in a central registry for studies at WMUH."
Allocation concealment (selection bias)	Unclear risk	Judgement: not described
Blinding of participants and personnel (performance bias)<br>: All outcomes	High risk	Quote: "All data on postoperative outcomes were collected and discussed by at least two surgeons who were not blinded to the allocation."
Blinding of outcome assessment	Low risk	Quote: "double blinded (investigator, outcome

(detection bias)<br>: Subjective outcomes		assessor)" †
Blinding of outcome assessment (detection bias)<br>: Objective outcomes	Low risk	Judgement: objective outcomes were unlikely affected by lack of blinding.
Incomplete outcome data (attrition bias)<br>: Overall mortality	Low risk	Judgement: all participants were included in the analysis
Incomplete outcome data (attrition bias)<br>: Postoperative complication	Low risk	Judgement: all participants were included in the analysis
Incomplete outcome data (attrition bias)<br>: Disease recurrence	Low risk	Judgement: all participants were included in the analysis
Incomplete outcome data (attrition bias)<br>: Cancer specific mortality	Unclear risk	Judgement: no information (not measured)
Incomplete outcome data (attrition bias)<br>: Postoperative mortality	Low risk	Judgement: all participants were included in the analysis
Incomplete outcome data (attrition bias)<br>: Anastomotic leakage	Low risk	Judgement: all participants were included in the analysis
Incomplete outcome data (attrition bias)<br>: Urinary dysfunction assessed by the ICIQ-UI and/or the IPSS	Unclear risk	Judgement: no information (not measured)
Incomplete outcome data (attrition bias)<br>: Sexual dysfunction assessed by the IIEF and/or the FSFI	Unclear risk	Judgement: no information (not measured)
Incomplete outcome data (attrition bias)<br>: Defecatory dysfunction assessed by the FIQL, the JWIS, the AWCS, and the GIQLI	Unclear risk	Judgement: 43/51 (84.3%) and 39/49 (80.0%) participants randomized to high ligation and low ligation were included in the analysis, respectively
Selective reporting (reporting bias)	High risk	Judgement: oncologic outcome in material and method section but it was not in result section
Other source of bias	Low risk	Judgement: Not detected

JWIS, Jorge-Wexner incontinence score, FIQL, fecal incontinence quality of life, ICIQ-UI, international consultation on incontinence questionnaire urinary incontinence, IPSS, international prostate symptom score, IIEF, international index of erectile function, FSFI, female sexual function index, AWCS, Agachan-Wexner constipation score, GIQLI, gastrointestinal quality of life index

† From study protocol

J. Niu 2016

Study characteristics	
Methods	**Study design:** Randomized controlled trial<br>**Setting/country:** single center/China<br>**Date when study was conducted:** March 2009 to March 2015
Participants	**Inclusion criteria**<br>• All of whom had preoperative colonoscopic pathology to confirm rectal cancer<br>**Exclusion criteria**<br>• Preoperative neoadjuvant chemotherapy<br>• Preoperative neoadjuvant radiotherapy<br><br>**Total number of participants randomly assigned:** 97<br><br>**High ligation**<br>• Number of all participant randomly assigned: 45<br>**Low ligation**<br>• Number of all participant randomly assigned: 52
Interventions	**Intervention:** high ligation<br>**Comparator:** low ligation<br>**Follow-up:** 36 months
Outcomes	**Outcomes**<br>• Anastomotic leakage<br>• Bleeding during operation<br>• Operation time<br>• Harvested lymph node<br>• Gas out<br>• Recurrence<br>• Metastasis
Funding sources	Not reported
Declarations of interest	None
Notes	**Protocol:** Not reported<br>**Language of publication:** Chinese

Risk of bias		
Bias	Authors' judgement	Support for judgement
Random sequence generation (selection bias)	Unclear risk	Judgement: not described
Allocation concealment (selection bias)	Unclear risk	Judgement: not described
Blinding of participants and personnel (performance bias)<br>: All outcomes	Unclear risk	Judgement: not described
Blinding of outcome assessment (detection bias)<br>: Subjective outcomes	Unclear risk	Judgement: not described
Blinding of outcome assessment (detection bias)<br>: Objective outcomes	Low risk	Judgement: objective outcomes were unlikely affected by lack of blinding.
Incomplete outcome data (attrition bias)<br>: Overall mortality	Unclear risk	Judgement: no information (not measured)
Incomplete outcome data (attrition bias)<br>: Postoperative complication	Unclear risk	Judgement: no information (not measured)

Incomplete outcome data (attrition bias)<br>: Disease recurrence	Unclear risk	Judgement: no information (not measured)
Incomplete outcome data (attrition bias)<br>: Cancer specific mortality	Unclear risk	Judgement: no information (not measured)
Incomplete outcome data (attrition bias)<br>: Postoperative mortality	Unclear risk	Judgement: no information (not measured)
Incomplete outcome data (attrition bias)<br>: Anastomotic leakage	Low risk	Judgement: all participants were included in the analysis
Incomplete outcome data (attrition bias)<br>: Urinary dysfunction assessed by the ICIQ-UI and/or the IPSS	Unclear risk	Judgement: no information (not measured)
Incomplete outcome data (attrition bias)<br>: Sexual dysfunction assessed by the IIEF and/or the FSFI	Unclear risk	Judgement: no information (not measured)
Incomplete outcome data (attrition bias)<br>: Defecatory dysfunction assessed by the FIQL, the JWIS, the AWCS, and the GIQLI	Unclear risk	Judgement: no information (not measured)
Selective reporting (reporting bias)	Unclear risk	Judgment: Study protocol was not identified
Other source of bias	Low risk	Judgement: Not detected

ICIQ-UI, international consultation on incontinence questionnaire urinary incontinence, IPSS, international prostate symptom score, IIEF, international index of erectile function, FSFI, female sexual function index, FIQL, fecal incontinence quality of life, JWIS, Jorge-Wexner incontinence score, AWCS, Agachan-Wexner constipation score, GIQLI, gastrointestinal quality of life index

K. Wang 2015

Study characteristics	
Methods	**Study design:** Randomized controlled trial<br>**Setting/country:** single center/China<br>**Date when study was conducted:** January 1 2013 to December 2013
Participants	**Inclusion criteria**<br>• Patients with rectal cancer undergoing low anterior resection<br>• R0 resection<br>• End-to-end anastomosis with a double anastomosis.<br>**Exclusion criteria**<br>• Bottom edge of tumor more than 15 cm from the anal verge<br>• Recurrence of rectal cancer or inability to perform radical surgery<br>• Clinical Tis and T1 stage (Japanese guidelines for colorectal cancer recommend low inferior mesenteric artery ligation for this group of patients)<br>• Preoperative radiotherapy<br>• Intraoperative protective stoma.<br><br>**Total number of participants randomly assigned:** 128<br><br>**High ligation**<br>• Number of all participant randomly assigned: 63<br>**Low ligation**<br>• Number of all participant randomly assigned: 65
Interventions	**Intervention:** high ligation<br>**Comparator:** low ligation<br>**Follow-up:** 12 months
Outcomes	**Primary outcomes**<br>• Bowel function (Wexner score of faecal incontinence, the number of bowel movements/d, the ability to distinguish defecation from excretion, the feeling of incomplete defecation within 15 min, the urgency of defecation, nocturnal defecation or noctunal incontinence, the use of antidiarrheal agents or laxatives, fecal incontinence quality of life score)<br>**Secondary outcomes**<br>• post-operative complication<br>• Anastomotic leakage<br>• Anastomotic stricture<br>• Urinary retention<br>• Oncological outcomes (number of harvested lymph node, number of metastatic lymph node, recurrence)
Funding sources	Not reported
Declarations of interest	None
Notes	**Protocol:** Not reported<br>**Language of publication:** Chinese

Risk of bias		
Bias	Authors' judgement	Support for judgement
Random sequence generation (selection bias)	Low risk	Quote: "applying the random number table method"
Allocation concealment (selection bias)	Unclear risk	Judgement: not described
Blinding of participants and personnel (performance bias)<br>: All outcomes	Unclear risk	Judgement: not described
Blinding of outcome assessment (detection bias)<br>: Subjective outcomes	Unclear risk	Judgement: not described
Blinding of outcome assessment	Low risk	Judgement: objective outcomes were unlikely

(detection bias)<br>: Objective outcomes		affected by lack of blinding.
Incomplete outcome data (attrition bias)<br>: Overall mortality	Unclear risk	Judgement: no information (not measured)
Incomplete outcome data (attrition bias)<br>: Postoperative complication	Low risk	Judgement: all participants were included in the analysis
Incomplete outcome data (attrition bias)<br>: Disease recurrence	Low risk	Judgement: all participants were included in the analysis
Incomplete outcome data (attrition bias)<br>: Cancer specific mortality	Unclear risk	Judgement: no information (not measured)
Incomplete outcome data (attrition bias)<br>: Postoperative mortality	Unclear risk	Judgement: no information (not measured)
Incomplete outcome data (attrition bias)<br>: Anastomotic leakage	Low risk	Judgement: all participants were included in the analysis
Incomplete outcome data (attrition bias)<br>: Urinary dysfunction assessed by the ICIQ-UI and/or the IPSS	Unclear risk	Judgement: no information (not measured)
Incomplete outcome data (attrition bias)<br>: Sexual dysfunction assessed by the IIEF and/or the FSFI	Unclear risk	Judgement: no information (not measured)
Incomplete outcome data (attrition bias)<br>: Defecatory dysfunction assessed by the FIQL, the JWIS, the AWCS, and the GIQLI	Unclear risk	Judgement: 53/63 (84.1%) and 58/65 (89.2%) participants randomized to high ligation and low ligation were included in the analysis, respectively (long-term)
Selective reporting (reporting bias)	Unclear risk	Judgement: study protocol was not identified
Other source of bias	Low risk	Judgement: Not detected

ICIQ-UI, international consultation on incontinence questionnaire urinary incontinence, IPSS, international prostate symptom score, IIEF, international index of erectile function, FSFI, female sexual function index, FIQL, fecal incontinence quality of life, JWIS, Jorge-Wexner incontinence score, AWCS, Agachan-Wexner constipation score, GIQLI, gastrointestinal quality of life index

L. Wu 2017

Study characteristics	
Methods	**Study design:** Randomized controlled trial<br>**Setting/country:** single center/China<br>**Date when study was conducted:** July 2014 to July 2016
Participants	**Inclusion criteria**<br>• Diagnosed with low rectal cancer without invasion or adhesion to other organs or structures<br>• Under the age of 70 years and able to tolerate laparoscopic surgery<br>**Exclusion criteria**<br>• Without severe cardiopulmonary disease, renal dysfunction, dyshepatia or metabolic disorders, without metastasis<br>• Without intestinal obstruction, perforation or gastroenteritis, never received radiotherapy or chemotherapy<br><br>**Total number of participants randomly assigned:** 96<br><br>**High ligation**<br>• Number of all participant randomly assigned: 50<br>**Low ligation**<br>• Number of all participant randomly assigned: 46
Interventions	**Intervention:** high ligation<br>**Comparator:** low ligation<br>**Follow-up:** not reported
Outcomes	**Outcomes**<br>• Intraoperative hemorrhage<br>• Operative time<br>• Anastomosis blood supply<br>• Third-level lymph node dissection<br>• If frequent defecation, difficult defecation and fecal incontinence occur after the surgery.
Funding sources	Not reported
Declarations of interest	None
Notes	**Protocol:** Not reported<br>**Language of publication:** Chinese

Risk of bias		
Bias	Authors' judgement	Support for judgement
Random sequence generation (selection bias)	Unclear risk	Judgement: not described
Allocation concealment (selection bias)	Unclear risk	Judgement: not described
Blinding of participants and personnel (performance bias)<br>: All outcomes	Unclear risk	Judgement: not described
Blinding of outcome assessment (detection bias)<br>: Subjective outcomes	Unclear risk	Judgement: not described
Blinding of outcome assessment (detection bias)<br>: Objective outcomes	Low risk	Judgement: objective outcomes were unlikely affected by lack of blinding.
Incomplete outcome data (attrition bias)<br>: Overall mortality	Unclear risk	Judgement: no information (not measured)
Incomplete outcome data (attrition bias)	Low risk	Judgement: all participants were included in the analysis

: Postoperative complication		
Incomplete outcome data (attrition bias)<br>: Disease recurrence	Unclear risk	Judgement: no information (not measured)
Incomplete outcome data (attrition bias)<br>: Cancer specific mortality	Unclear risk	Judgement: no information (not measured)
Incomplete outcome data (attrition bias)<br>: Postoperative mortality	Unclear risk	Judgement: no information (not measured)
Incomplete outcome data (attrition bias)<br>: Anastomotic leakage	Low risk	Judgement: all participants were included in the analysis
Incomplete outcome data (attrition bias)<br>: Urinary dysfunction assessed by the ICIQ-UI and/or the IPSS	Unclear risk	Judgement: no information (not measured)
Incomplete outcome data (attrition bias)<br>: Sexual dysfunction assessed by the IIEF and/or the FSFI	Unclear risk	Judgement: no information (not measured)
Incomplete outcome data (attrition bias)<br>: Defecatory dysfunction assessed by the FIQL, the JWIS, the AWCS, and the GIQLI	Unclear risk	Judgement: no information (not measured)
Selective reporting (reporting bias)	Unclear risk	Judgement: study protocol was not identified
Other source of bias	Low risk	Judgement: Not detected

ICIQ-UI, international consultation on incontinence questionnaire urinary incontinence, IPSS, international prostate symptom score, IIEF, international index of erectile function, FSFI, female sexual function index, FIQL, fecal incontinence quality of life, JWIS, Jorge-Wexner incontinence score, AWCS, Agachan-Wexner constipation score, GIQLI, gastrointestinal quality of life index

M. Zhou 2018

Study characteristics	
Methods	**Study design:** prospective randomized controlled trial<br>**Setting/country:** single center/China<br>**Date when study was conducted:** October 2015 to June 2016
Participants	**Inclusion criteria**<br>• Patients with rectal cancer who were confirmed to have<br>complete resection of the primary tumor and no distant metastasis, 2 to 15 cm from the anus after preoperative examination<br>• Patients aged 18 to 75 years old who could undergo laparoscopic surgery<br>• No obvious contraindications to surgery<br>**Exclusion criteria**<br>• Patients who underwent emergency surgery for various<br>reasons, excluding those who underwent postoperative emergency surgery due to complications<br>• Patients with combined obstruction, or those who underwent intestinal stenting for obstruction<br>• Patients with previous history of abdominal surgery<br>• Patients who did not undergo intraoperative rectal resection for various reasons, excluding those who underwent intermediate open surgery<br>• Patients who underwent preoperative radiotherapy<br>• Patients with multiple primary malignant tumors of the colorectum.<br><br>**Total number of participants randomly assigned:** 104<br><br>**High ligation**<br>• Number of all participant randomly assigned: 52<br>**Low ligation**<br>• Number of all participant randomly assigned: 52
Interventions	**Intervention:** high ligation<br>**Comparator:** low ligation<br>**Follow-up:** 1 month
Outcomes	**Outcomes**<br>• Intraoperative conditions: operation time, intraoperative bleeding, accuracy rate of vascular ligation, intermediate open rate, and prophylactic stoma rate<br>• Postoperative pathology: tumor staging and staging rate before and after surgery, number of lymph node dissection and positive lymph node rate<br>• Postoperative conditions: first anal venting time, hospitalization time, total amount of abdominal drainage and abdominal drainage tube stay time, urinary catheter<br>• Postoperative complications: including urinary dysfunction, anastomotic bleeding and anastomotic leak and the re-hospitalization rate and death rate within 30 days after surgery.
Funding sources	Guangzhou Important Special Program of Heahh Medicine Cooperation and Innovation (201604020005)
Declarations of interest	None
Notes	**Protocol:** Not reported<br>**Language of publication:** Chinese

Risk of bias		
Bias	Authors' judgement	Support for judgement
Random sequence generation (selection bias)	Low risk	Quote: "According to the principle of complete randomization"
Allocation concealment (selection bias)	Unclear risk	Judgement: not described
Blinding of participants and personnel (performance bias)<br>: All outcomes	Unclear risk	Judgement: not described

Blinding of outcome assessment (detection bias)<br>: Subjective outcomes	Unclear risk	Judgement: not described
Blinding of outcome assessment (detection bias)<br>: Objective outcomes	Low risk	Judgement: objective outcomes were unlikely affected by lack of blinding.
Incomplete outcome data (attrition bias)<br>: Overall mortality	Unclear risk	Judgement: no information (not measured)
Incomplete outcome data (attrition bias)<br>: Postoperative complication	Low risk	Judgement: all participants were included in the analysis
Incomplete outcome data (attrition bias)<br>: Disease recurrence	Unclear risk	Judgement: no information (not measured)
Incomplete outcome data (attrition bias)<br>: Cancer specific mortality	Unclear risk	Judgement: no information (not measured)
Incomplete outcome data (attrition bias)<br>: Postoperative mortality	Low risk	Judgement: all participants were included in the analysis
Incomplete outcome data (attrition bias)<br>: Anastomotic leakage	Low risk	Judgement: all participants were included in the analysis
Incomplete outcome data (attrition bias)<br>: Urinary dysfunction assessed by the ICIQ-UI and/or the IPSS	Unclear risk	Judgement: no information (not measured)
Incomplete outcome data (attrition bias)<br>: Sexual dysfunction assessed by the IIEF and/or the FSFI	Unclear risk	Judgement: no information (not measured)
Incomplete outcome data (attrition bias)<br>: Defecatory dysfunction assessed by the FIQL, the JWIS, the AWCS, and the GIQLI	Unclear risk	Judgement: no information (not measured)
Selective reporting (reporting bias)	Unclear risk	Judgement: study protocol was not identified
Other source of bias	Low risk	Judgement: Not detected

ICIQ-UI, international consultation on incontinence questionnaire urinary incontinence, IPSS, international prostate symptom score, IIEF, international index of erectile function, FSFI, female sexual function index, FIQL, fecal incontinence quality of life, JWIS, Jorge-Wexner incontinence score, AWCS, Agachan-Wexner constipation score, GIQLI, gastrointestinal quality of life index
