# Supplementary material for: High Versus Low Ligation of the Inferior Mesenteric Artery in Colorectal Cancer Surgery: A Systematic Review and Meta-Analysis
Source: Medicina (Kaunas). 2022 Aug 23;58(9):1143. doi: 10.3390/medicina58091143 (PMC9506533; doi:10.3390/medicina58091143)
Supplement: Supplementary file 1 [file medicina-58-01143-s001.zip › supplementary_file_5_contact_detail.pdf]

Table S5. Details of contact with correspondents of included trials.

Study name	Date the author contacted	Date the author provided data	Short summary the data provided by the authors
Feng 2021	7 June 2021	15 June 2021	Postoperative mortality, Postoperative complications, random sequence generation, allocation concealment. Checking whether a published abstract is from the study of Feng et al
Kruszewshi 2021	22 June 2021	22 June 2021	Informed us to publish full-text article on July 2021
	23 July 2021	3 Augest 2021	Each number of patients of high ligation and low ligation analyzed for overall survival, disease-free survival, and cancer-specific survival, blinding of participant and personnel.
