# Supplementary material for: High Versus Low Ligation of the Inferior Mesenteric Artery in Colorectal Cancer Surgery: A Systematic Review and Meta-Analysis
Source: Medicina (Kaunas). 2022 Aug 23;58(9):1143. doi: 10.3390/medicina58091143 (PMC9506533; doi:10.3390/medicina58091143)
Supplement: Supplementary file 1 [file medicina-58-01143-s001.zip › supplementary_file_6_exc_study.pdf]

Table S6. Characteristics of excluded studies

**Study**	**Reason for exclusion**
Akagi 2020 (1 full-text article, 1 conference abstract)	Irrelevant study design (retrospective review using data collected prospectively for other study)
Komen 2011	Irrelevant study design (non-randomization)
Samalavicius 2018	Irrelevant study design (non-randomization)
NCT03520088	Irrelevant intervention and comparator (Inferior mesenteric artery dissection first versus inferior mesenteric vein dissection first)
